# Palmitoyl Acyltransferase Zdhhc17 Promotes Functional Recovery After Spinal Cord Injury by Targeting the Nuclear Transport Factors Kpna2 and Ipo9

**DOI:** 10.1002/advs.77724

**Published:** 2026-09-11

**Authors:** Meixuan Chen, Huan Chen, Sining Ding, Fan Liu, Xiaofei Zheng, Yuan Wang, Ruitu Tian, Huan Li, Peilin Liu, Le Hu, Bin Liu, Limin Rong, Mangmang Li

**Affiliations:** ^1^ Guangdong Provincial Key Laboratory of Bone and Joint Degeneration Diseases Department of Cell Biology School of Basic Medical Sciences Southern Medical University Guangzhou China; ^2^ Department of Spine Surgery The Third Affiliated Hospital of Sun Yat‐Sen University Guangzhou China; ^3^ Guangdong Provincial Center for Engineering and Technology Research of Minimally Invasive Spine Surgery Guangzhou China; ^4^ Guangdong Provincial Center for Quality Control of Minimally Invasive Spine Surgery Guangzhou China

**Keywords:** axon, cell biology, excitotoxicity, interactome, neuroprotection, oxidative stress, palmitoylation, protein palmitoylation, spinal cord injury

## Abstract

In adult mammals, poor functional recovery after spinal cord injury (SCI) is largely due to the very limited capacity to reconstruct damaged neural connections together with neuronal loss. Here, we identify the neuroprotective role of Zdhhc17 as a palmitoyl acyltransferase (PAT) following SCI. Neuron‑specific Zdhhc17 overexpression in vitro and in vivo markedly enhances axon regeneration and functional recovery after SCI in a PAT‐activity‐dependent manner. Interactome and palmitoylation analyses in cortical neurons identify the karyopherins Kpna2 and Ipo9 as previously unrecognized Zdhhc17 substrates. SCI markedly reduces Kpna2 and Ipo9 protein levels, whereas Zdhhc17‑mediated palmitoylation stabilizes them by suppressing their ubiquitin‑dependent degradation. Functionally, neuronal overexpression of Kpna2 or Ipo9 mimics the therapeutic effects of Zdhhc17, and co‑expression of Zdhhc17 with Ipo9, but not Kpna2, further augments SCI repair. Using H_2_O_2_‑induced oxidative stress and glutamate‑induced excitotoxicity models, we further show that Zdhhc17 promotes neuronal survival and activates transcription of intrinsic pro‑regenerative genes after injury. These findings suggest that the Zdhhc17‐Kpna2/Ipo9 axis is a novel pharmacological target for SCI treatment.

## Graphical abstract

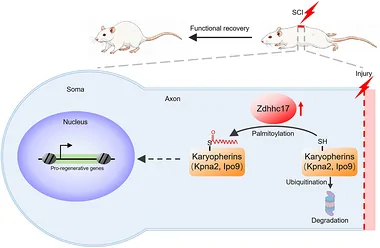

## 1 Introduction

In adult mammals, the failure of functional recovery after spinal cord injury (SCI) is largely due to the limited restoration of neural circuits [1]. By contrast, injury to peripheral nerves is followed by robust regeneration, which requires the induction of widespread transcriptome changes in injured neurons. Considerable progress has been made in recent years in understanding intrinsic signaling pathways that trigger the regenerative response following injury in the peripheral nervous system (PNS). However, because the central nervous system (CNS) differs markedly from the PNS, with a highly inhibitory extracellular environment and a much more limited intrinsic regenerative capacity of CNS neurons, the regenerative mechanisms defined in the PNS are not directly transferable to CNS injury [2, 3].

Protein S‑acylation is a post‑translational modification in which long‑chain fatty acids are attached via a thioester bond to specific cysteine residues on target proteins [4]. Because palmitate is the most abundant and commonly used fatty acid in this context, S‑acylation is often referred to as S‑palmitoylation. In mammalian cells, S‑palmitoylation is mainly catalyzed by the zinc finger DHHC‑containing palmitoyl S‑acyltransferase (PAT) family, whose members share a conserved DHHC (Asp‑His‑His‑Cys) catalytic motif [5]. Among the 23 members of this family, Zdhhc17, also known as Huntingtin interacting protein 14 (HIP14), was the first neuronal PAT identified to palmitoylate huntingtin (HTT) [6, 7]. More recent work has revealed that Zdhhc17 exerts both PAT‑dependent and PAT‑independent functions in neurons, playing key regulatory roles in neuronal development, synaptic plasticity, and axon growth [8, 9, 10]. However, in the context of CNS injury, particularly SCI, our understanding of Zdhhc17 remains very limited. Yang et al. reported a PAT‑independent role of Zdhhc17 in CNS neuronal death by regulating JNK activation [11]. Using an optic nerve crush (ONC) model, Niu et al. further showed that Zdhhc17 palmitoylates DLK and NMNAT2, two well‑known regulators of soma death and distal axon integrity, respectively, thereby coordinating the post‑injury neuronal response in retinal ganglion cells [12]. However, whether Zdhhc17 contributes to SCI repair and, if so, through which molecular mechanisms, has not yet been investigated.

In injured neurons, successful regeneration depends on efficient transmission of injury signals to the nucleus, activation of regeneration‐associated gene expression, and subsequent synthesis and transport of pro‐regenerative mRNAs and proteins. Nucleocytoplasmic transport, encompassing both nuclear import and export, is a central node in these processes. Karyopherins (Kaps) form the core machinery of this transport system. By recognizing nuclear localization signals (NLSs) and nuclear export signals (NESs) on cargo proteins, members of the importin‐α (KPNA) and karyopherin‐β (KPNB) subfamilies act as a highly regulated “molecular conveyor belt” that mediates bidirectional traffic between cytoplasm and nucleus [13]. The contribution of Kaps to nerve injury responses has been investigated mainly in peripheral sensory neurons. The most comprehensive insights come from a series of studies by Mike Fainzilber's laboratory, which identified a Kpnb1‐centered pathway in retrograde injury signaling [14, 15, 16, 17]. Importin‐α family members are constitutively present in axons of both intact and injured nerves. After lesion, local axonal translation of Kpnb1 and RanBP1 enables assembly of importin‐α/β heterodimers on dynein, thereby permitting importin‐binding cargo proteins—such as the pro‐regenerative transcription factor STAT3 [15]—to be imported into the nucleus to drive a regenerative transcriptional program. Disrupting this system, either by Kpnb1 knockout [16], by competition at the NLS‐binding pocket on importins [14], or by perturbing the RanGTPase system [17], attenuates injury‐induced transcriptional responses and functional recovery. Interestingly, key elements of this importin‐dependent retrograde injury signaling machinery have also been implicated in central neuronal regeneration [18, 19]. Of note, the potential roles and mechanisms of karyopherin palmitoylation in neuronal injury repair have not yet been explored.

Our pilot experiments revealed that Zdhhc17 protein levels are markedly reduced in a rat model of incomplete SCI and are partially restored following mesenchymal stem cell transplantation, suggesting that Zdhhc17 is important for maintaining normal spinal cord circuitry. Here, using a neuron‑specific adeno‑associated virus (AAV) expression system, we demonstrate that Zdhhc17 effectively promotes functional recovery after SCI in a PAT‐dependent manner. Proteomic analysis of the Zdhhc17 interactome in cortical neurons revealed interactions with numerous karyopherin family members. We then identified Kpna2 and Ipo9 as new substrates of Zdhhc17, whose protein, but not mRNA, levels change in parallel with Zdhhc17. Furthermore, Zdhhc17‑mediated palmitoylation suppresses their ubiquitin‑dependent degradation. Functional analyses in vitro and in vivo further revealed that Kpna2 and Ipo9 contribute to axon regeneration and SCI repair. Using neuronal models of oxidative stress and excitotoxicity, we found that Zdhhc17 reduces neuronal cell death and enhances the transcription of a broad set of pro‑regenerative genes. Taken together, our study highlights Zdhhc17‑mediated palmitoylation of Kaps as a potential therapeutic target for promoting neuronal survival/regeneration after SCI.

## 2 Materials and Methods

### 2.1 Ethics Statement

All animal experiments were conducted in accordance with the protocols approved by the Institutional Animal Care and Use Committee (IACUC) of Southern Medical University (Approval No. SMU202506019).

### 2.2 Plasmid Construction

For gene overexpression, the coding sequences of rat *Zdhhc17*, *Kpna2*, and *Ipo9* were PCR‐amplified from rat brain cDNA using gene‐specific primers (Table S1) and cloned into pLVX‐IRES‐puro (632183, Clontech) or pAAV‐hsynI (99126, Addgene) via homologous recombination (C115‐02, Vazyme). For gene knockdown, five shRNAs targeting each of Zdhhc17, Kpna2, and Ipo9 were individually designed and ligated into pLKO.1‐EGFP using T4 DNA ligase (M0202V, Biolabs). Primer sequences and plasmid details are provided in Tables S1 and S2, respectively.

### 2.3 Cell Culture

HEK293T cells (ATCC) were cultured in DMEM (10‐013‐CVRC, Corning) with 10% FBS (SERANA, Germany), 1% NEAA (N1250, Solarbio), and 1% penicillin‐streptomycin (30‐002‐Cl, Corning) at 37°C with 5% CO_2_.

Primary cortical neurons were isolated from embryonic day 18–19 (E18‐19) Sprague–Dawley rats as described previously [20]. Briefly, embryos were collected in ice‐cold dissection solution (DMEM supplemented with 1 mM sodium pyruvate (11360070, Gibco) and 10 µg/mL gentamicin (G1264, Sigma–Aldrich)). Cortices were digested at 37°C for 1 h in pre‐warmed digestion buffer (dissection solution containing 4.2 mg/mL papain (KEH, 9001‐73‐4) and 10 mg/mL DNase I (Roche, 4716728001)). Tissue was gently triturated in stop solution (dissection solution supplemented with 10% FBS) and centrifuged at 600×g for 6 min at 4°C. Cells were resuspended in neuronal complete medium (Neurobasal‐A medium (21103‐049, Gibco), 2% B27 (17504044, Thermo Fisher Scientific), 1 mM sodium pyruvate, 0.5 mM L‐glutamine (35050‐061, Gibco), and 10 µg/mL gentamicin), filtered through a 70 µm strainer (352350, Falconc), and seeded onto poly‐D‐lysine‐coated plates (HY‐139201A, MedChemExpress). At 7 days in vitro (DIV7), half of the culture medium was replaced with fresh neuronal complete medium.

### 2.4 Virus Generation

Lentivirus was generated as previously described [21]. 239T cells were transfected with 12 µg total DNA per 10 cm dish (pCMV‐ΔR8, pCMV‐VSV‐G, and lentiviral expression vector at a 4:2:6 ratio) using PEI (78BioPEI25000, BIOHUB) at a 5:1 PEI:DNA ratio. The medium was replaced at 10 h post‐transfection. Viral supernatants were collected at 48 and 72 h, filtered (0.45 µm, SLHV033NS, Merck), and ultracentrifuged at 25 500 rpm for 2 h at 4°C. Viral particles were resuspended in 1 mL neuronal medium overnight at 4°C, aliquoted, and stored at −80°C.

Adeno‐associated virus (AAV) was generated as previously described [22]. Lenti‐X 293T cells in 15 cm dishes were transfected with pHelper, pUCmini‐iCAP‐PHP.eB, and pAAV vector at a 1:2:1 ratio (40 µg total DNA per dish) using PEI at a 5:1 PEI:DNA ratio. The medium was replaced with 5% FBS‐DMEM at 8 h post‐transfection. AAV‐containing medium was collected at 72 and 120 h, and cells were additionally harvested at 120 h. AAV particles were released from cells by Salt‐active nuclease (SAN; ArcticZymes, 70910‐202) digestion, pooled with those in the supernatant, and precipitated with PEG8000 (Sigma, 89510–1KG‐F). The particles were further purified by iodixanol density gradient ultracentrifugation at 350 000×g for 2 h at 18°C, then concentrated to 500 µL in DPBS using Amicon Ultra‐15 centrifugal filter devices (Millipore, UFC910024). Viral titer was determined by qPCR. Purified AAV was adjusted to 1.25 × 10^14^ vg/mL, and 40 µL (5 × 10^1^ ^2^ vg) was intrathecally administered to rats one week before SCI.

### 2.5 Subacute Incomplete SCI Model in Rats

SCI was induced using a well‐established compression model as previously described [23]. Briefly, adult female Sprague–Dawley rats (220–250 g) were anesthetized with an intraperitoneal injection of 1% sodium pentobarbital (25 mg/kg; Fluka, Sigma–Aldrich). Following laminectomy at the T9–T11 vertebrae, a 60 g aneurysm clip (Sugita Corporation, Tokyo, Japan) was applied at the T10 for 60 s. Postoperatively, rats received cefazolin sodium (50 000 units/kg/day; Jusheng Pharmaceutical Co., Ltd., China) for 5 days, and manual bladder expression was performed twice daily.

### 2.6 Behavioral Experiments

Hindlimb motor function was evaluated weekly by two blinded observers using three tests. Each test was performed in triplicate per animal, and the average value was calculated.

#### 2.6.1 Basso, Beattie, and Bresnahan (BBB) Locomotor Rating Scale

Rats were placed in a circular open field (90 cm diameter) for ≥ 5 min. Locomotor function was scored on a 0–21 scale according to standard BBB criteria.

#### 2.6.2 Beam Walking Test

Motor coordination was assessed by recording the ability of rats to traverse a narrow wooden beam (150 cm length, 2.5 cm width, 50 cm above floor). The number of paw slips and time to cross were scored on a 0–7 scale.

#### 2.6.3 Rivlin Test

Hindlimb support capacity was measured using an inclined plane with a textured rubber surface. Rats were placed perpendicular to the incline, and the maximum angle at which they could maintain position for 5 s without sliding was recorded. The angle was increased in 5° increments.

#### 2.6.4 Swimming Test

Rats were placed in a rectangular Plexiglas chamber (150 × 15 × 40 cm) filled with tap water (depth 20–23 cm, temperature 28°C–32°C) [24]. A platform was placed at the right end of the chamber to guide the rats to swim from left to right. Swimming performance was recorded at 4 weeks post‐injury.

### 2.7 Motor Evoked Potential (MEP) Recording

MEPs were recorded from six rats per group to assess motor pathway integrity. Under anesthesia, a cranial window (3 mm diameter) was made over the right motor cortex. A bipolar stimulating electrode was inserted 0.5 mm into the cortex, and a recording electrode was placed on the contralateral sciatic nerve. MEPs were evoked by a single square‐wave pulse (10 V, 1 ms pulse width, 50 ms delay). Amplitude (mV) and latency (ms) were measured using a BL‐420A/F data acquisition system.

### 2.8 Biotinylated Dextran Amine (BDA) Tracing

BDA tracing was performed to evaluate corticospinal tract (CST) axonal regeneration after SCI [25]. Briefly, BDA (10 000 MW, 10% in PBS; D1956, Thermo Fisher Scientific, USA) was stereotactically injected into the bilateral sensorimotor cortex at 24 sites (0.25 µL/site) according to established coordinates relative to bregma at two weeks post‐injury (wpi). Two weeks later, transverse sections of the spinal cord at the T5 and T10 levels were stained with Alexa Fluor 488‐conjugated streptavidin (A‐11055, Thermo Fisher Scientific, USA) to evaluate BDA‐labeled CST axons.

### 2.9 Western Blotting (WB)

Cells or spinal cord tissues were lysed in RIPA buffer (150 mM NaCl, 50 mM Tris‐HCl pH 8.0, 5 mM EDTA, 1% Triton X‐100, 0.1% SDS, 1% sodium deoxycholate) supplemented with protease inhibitors (5 µg/mL leupeptin, 5 µg/mL pepstatin, 10 µg/mL aprotinin; Roche). Soluble proteins were collected by centrifugation (13 000×g, 10 min, 4°C), and protein concentrations were measured by BCA assay (23227, Thermo Scientific). Equal amounts (50–100 µg) of protein were separated by SDS‐PAGE, transferred to nitrocellulose membranes (1060001, GE), blocked in 5% non‐fat milk/PBST, and incubated with primary antibodies overnight at 4°C, followed by HRP secondary antibodies for 1 h at RT. Signals were detected using ECL (ANEL105001E, PerkinElmer) and quantified with ImageJ. Antibodies are listed in Table S3.

### 2.10 Immunofluorescence (IF) Staining

For tissue sections, frozen slides were thawed to room temperature (RT). For cultured cells, cells were fixed with 4% paraformaldehyde. Samples were washed three times with PBS and blocked with blocking buffer (2% BSA and 1% Triton X‐100 in PBS) for 2 h at RT. Primary antibodies were applied overnight at 4°C. After three PBS washes, samples were incubated with fluorescence‐conjugated secondary antibodies for 1 h at RT in the dark. Nuclei were counterstained with DAPI for 15 min. After three additional PBS washes, slides were mounted with 50% glycerol and sealed with nail polish. Images were acquired using a confocal microscope. Antibodies are listed in Table S3.

### 2.11 Hematoxylin and Eosin (H&E) Staining

Frozen sections were dried at 37°C for 15 min, washed three times with PBS, and stained using an H&E Staining Kit (C0105S, Beyotime) according to the manufacturer's instructions. Sections were then dehydrated in ethanol, cleared in xylene, and mounted with neutral balsam.

### 2.12 RNA Extraction and Quantitative Real‐Time PCR (qPCR)

Total RNA was extracted from cells using TRIzol. cDNA synthesized from 1 µg of total RNA was diluted 10‐fold for qPCR using SYBR Green Master Mix (11203ES08, Yeasen) on a StepOnePlus system (Applied Biosystems). 28S rRNA was used as an internal control, and relative expression was calculated using the 2^−^^ΔΔCt^ method. Primer sequences are listed in Table S1.

### 2.13 Neuronal Scratch Assay

Primary cortical neurons seeded at 3 × 10^5^ cells/well in 24‐well plates were transduced with lentivirus on DIV7. At 3 days post‐transduction, a scratch injury was made using a sterile 10 µL pipette tip. Debris and detached cells were removed by half‐medium exchange. 50 ng/mL recombinant human BDNF (rhBDNF; Z03208, Genscript) treatment was used as a positive control. At 4 days post‐scratch, axon regeneration was assessed by anti‐βIII‐tubulin immunofluorescence.

### 2.14 Immunoprecipitation and Mass spectrometry (IP‐MS)

For immunoprecipitation, 2 mg of soluble proteins from primary cortical neurons at DIV14 was incubated with 2 µg of rabbit anti‐Zdhhc17 antibody (30022‐1‐AP, Proteintech) or normal rabbit IgG (2729S, CST) overnight at 4°C. Protein‐antibody complexes were captured using protein G Sepharose beads (17‐0618‐01, GE) and eluted in SDS sample buffer with 5% β‐mercaptoethanol. Proteins were separated by SDS‐PAGE and silver‐stained using a mass spectrometry‐compatible kit (24600, Thermo Scientific). Entire gel lanes were excised and analyzed by LC‐MS/MS. Raw data were processed with MaxQuant (version 1.6.14) and searched against the appropriate protein database.

### 2.15 Acyl‐Resin Assisted Capture (Acyl‐RAC) Assay

Palmitoylation was assessed as previously described [26]. 1 mg of protein was diluted in blocking buffer (100 mM HEPES, 1 mM EDTA, 2.5% SDS, 5% MMTS, pH 7.5) to 2 mg/mL and incubated overnight at 4°C. Proteins were precipitated with 1.5 mL pre‐chilled acetone at −20°C for 20 min, centrifuged at 5000×g for 10 min at 4°C, and washed five times with 1 mL pre‐chilled 70% acetone (15 000×g, 5 min, 4°C). Proteins resuspended in 600 µL binding buffers (100 mM HEPES, 1 mM EDTA, 1% SDS, pH 7.5) were incubated with 20 µL of thiol‐affinity resin (20511ES08, Yeasen) and 40 µL of freshly prepared 2 M hydroxylamine (pH 7.5, ACMEC‐5470111) for 2–4 h at RT. After centrifugation (2000 rpm, 3 min), the resin was washed with binding buffer 3–5 times. Bound proteins were eluted in 20 µL of SDS sample buffer and analyzed by WB.

### 2.16 Cycloheximide (CHX) Chase Assay

HEK293T cells were seeded in six‐well plates at 0.8 × 10^6^ cells per well one day before treatment. CHX (66‐81‐9, MCE) was added at a final concentration of 100 µg/mL, and cells were harvested at 0, 4, 8, and 12 h for WB analysis.

### 2.17 MG132 Treatment and Ubiquitination Assay

HEK293T cells seeded in 6 cm dishes at 2.5 × 10^6^ cells per dish were treated with MG132 (A181909, AmBeed) at a final concentration of 10 µM for 10 h. One mg of total protein from cells was incubated with 1 µg of MYC‐tag antibody (M20002M, Abmart) overnight at 4°C, followed by 20 µL of Protein A/G agarose beads (L00664‐5, GeneScript) for 2–4 h. Immunoprecipitates were eluted in SDS sample buffer and analyzed by WB using ubiquitin antibody (3936T, CST).

### 2.18 Lactate Dehydrogenase (LDH) Assay

Primary cortical neurons seeded at 3 × 10^5^ cells/well in 24‐well plates on DIV10 were treated with 300 µM H_2_O_2_ (7722‐84‐1, Merck) [27] or 4 µM glutamate (A749276, AmBeed) [28] for 24 h. Supernatants were collected, centrifuged at 1500 rpm for 5 min, and 100 µL of each supernatant was used for detection. 50 µL of LDH working solution prepared according to the manufacturer's instructions (Beyotime, C0019S) was added to each sample, vortexed, and incubated at 37°C for 30 min in the dark with shaking. Absorbance was measured at 450 nm. The LDH release rate was calculated as follows: (OD‐experimental – OD‐blank) / (OD‐maximal release – OD‐blank) × 100%.

### 2.19 Statistical Analysis

Data are presented as mean ± SEM. Statistical analyses were performed using GraphPad Prism 9.0. Comparisons between two groups were analyzed by two‐tailed unpaired Student's *t*‐test. For multi‐group comparisons, one‐way or two‐way ANOVA was used, followed by Bonferroni's post hoc test for selected pairwise comparisons based on our predefined biological hypotheses. Significance levels were defined as follows: ns, not significant; ^*^ *p* < 0.05, ^**^ *p* < 0.01, ^***^ *p* < 0.001, ^****^ *p* < 0.0001.

## 3 Results

### 3.1 Neuron‐Specific Restoration of Zdhhc17 Promotes Functional Recovery After SCI

**FIGURE 1:**
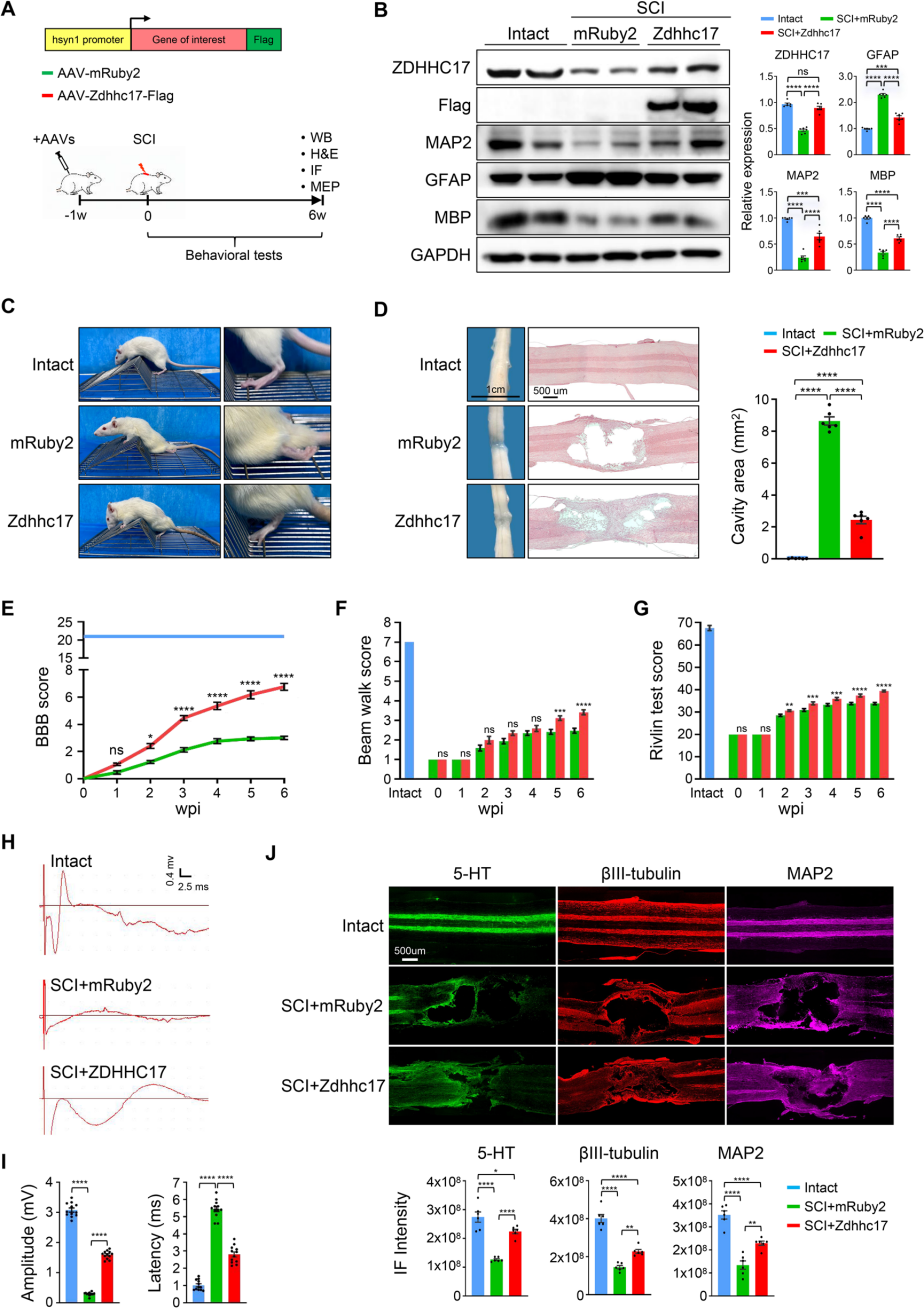
Neuron‐specific restoration of Zdhhc17 promotes functional recovery after SCI. (A) Schematic of the experimental design. AAV‐mRuby2 served as the negative control. (B) WB analysis of Flag‐tagged Zdhhc17, MAP2, GFAP, MBP, and GAPDH in the lesion sites from different groups. Right panels show quantification of the indicated protein expression levels. The color key is consistent across all panels. Data are presented as the mean ± SEM (*n* = 3 per group). ns, not significant; ^**^ *p* < 0.01; ^***^ *p* < 0.001; ^****^ *p* < 0.0001 by one‐way ANOVA. (C) Representative images of rats with different treatments 6 weeks after injury. (D) Representative images of the gross morphology of spinal cords at the T5‐L2 levels and H&E staining of spinal cords at the lesion sites. Right panel shows quantification of the lesion area. Data are presented as the mean ± SEM (*n* = 6 per group). ^****^ *p* < 0.0001 by one‐way ANOVA. (E–G) Behavioral assessments of functional recovery following SCI in groups with indicated treatments, including BBB score (E), beam walk score (F), and Rivlin test (G). Data are presented as mean ± SEM (*n* = 12/group/time point). ns, not significant; ^*^ *p* < 0.05; ^**^ *p* < 0.01; ^***^ *p* < 0.001; ^****^ *p* < 0.0001 by two‐way ANOVA comparing AAV‐mRuby2 with other groups. (H) Representative MEP recordings from rats with different treatments at 6 wpi. (I) Quantification of MEP amplitude and latency. Data are presented as mean ± SEM (*n* = 6 per group). ^****^ *p* < 0.0001 by one‐way ANOVA. (J) Representative IF images of spinal cord coronal sections around injured site immunostained with antibodies against 5‐HT, βIII‐tubulin, and MAP2 in rats with indicated treatments at the end of the experiment. Lower panels, quantification of the indicated immunoreactive intensity. The color key is consistent across all panels. Data are presented as mean ± SEM (*n* = 6 per group). ^*^ *p* < 0.05; ^**^ *p* < 0.01; ^****^ *p* < 0.0001 by one‐way ANOVA.

Given that SCI caused a significant reduction in Zdhhc17 expression, particularly in spinal neurons (Figure 1B and Figure S1), we sought to determine whether Zdhhc17 contributes to functional repair. To this end, we used an AAV‐based neuron‐specific expression system driven by the human synapsin‐1 (hSyn1) promoter to overexpress Flag‐tagged Zdhhc17 in adult rat spinal neurons, followed by T9–T11 contusive SCI (Figure 1A). WB analysis of spinal cord tissue 6 weeks post‐injury (wpi) showed that the SCI‐induced reductions in Zdhhc17 and MAP2 in the AAV‐mRuby2 control group were substantially restored in rats receiving AAV‑Zdhhc17‑Flag (Figure 1B). Observation of hindlimb movement patterns revealed that SCI rats typically stood with the dorsal surface of the hind paws contacting the ground, indicative of severe motor impairment. In contrast, rats with Zdhhc17 overexpression (OE) were able to support themselves with proper plantar placement of the hind paws (Figure 1C). In line with this, the beneficial effects of Zdhhc17 were further supported by gross morphology and preservation of spinal parenchymal integrity (Figure 1D), by multiple behavioral assessments including BBB scores (Figure 1E), beam walk performance (Figure 1F), and Rivlin test scores (Figure 1G), and by improved hindlimb motor evoked potentials (MEPs) (Figure 1H,I). Finally, Zdhhc17 robustly promoted the regrowth of nerve fibers within the epicenter, as demonstrated by increased signal intensity of serotonin (5‑HT), βIII‑tubulin, and MAP2 (Figure 1J). Together, these findings demonstrate that neuron‐specific restoration of Zdhhc17 significantly enhances structural and functional recovery after SCI.

### 3.2 Zdhhc17 Enhances Axon Regeneration In Vitro in a PAT‑Dependent Manner

**FIGURE 2:**
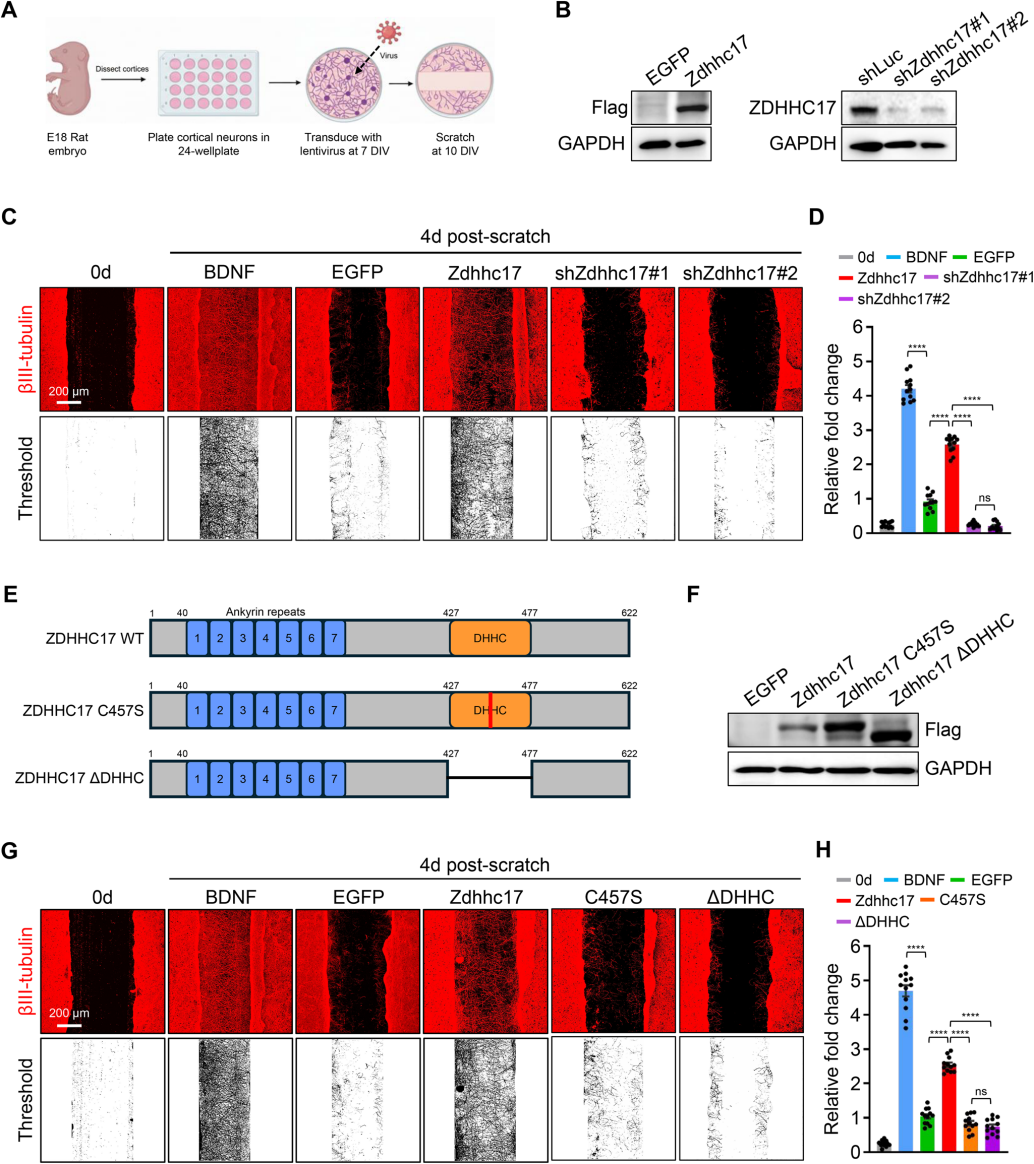
Zdhhc17 enhances axon regeneration in vitro in a PAT‐dependent manner. (A) Schematic illustration of the scratch assay. (B) WB analysis of ZDHHC17 protein levels in cortical neurons under Zdhhc17 overexpression and shRNA‐mediated knockdown conditions. (C) Representative images of scratch‑injured cortical cultures with Zdhhc17 overexpression (OE) or knockdown (KD), immunostained for βIII‑tubulin, alongside the corresponding ImageJ‑thresholded images. Recombinant human BDNF served as a positive control. (D) Quantification of βIII‐tubulin intensity in the scratched area upon various treatments in (C). Data are presented as mean ± SEM (*n* = 12). ns, not significant; ^****^ *p* < 0.0001 by one‐way ANOVA. (E) Schematic illustration of wild‑type (WT) and mutant Zdhhc17 proteins (ZDHHC17 C457S and ZDHHC17ΔDHHC). (F) WB analysis of Flag‑tagged WT and mutant Zdhhc17 in cortical neurons. (G) Representative images of scratch‑injured cortical cultures overexpressing WT Zdhhc17 or enzymatic mutants, immunostained for βIII‑tubulin, with corresponding ImageJ‑thresholded images. (H) Quantification of βIII‐tubulin intensity in the scratched area upon various treatments in (G). Data are presented as mean ± SEM (*n* = 12). ns, not significant; ^****^ *p* < 0.0001 by one‐way ANOVA.

To test whether Zdhhc17 promotes neurite regeneration after injury, we performed an in vitro scratch assay. Rat cortical neurons derived from embryonic day 18 (E18) brains were transduced with lentiviruses expressing Flag‑tagged Zdhhc17 or shRNAs against Zdhhc17 (shZdhhc17#1 and #2) (Figure 2B), and were mechanically injured with a pipette tip (Figure 2A). Axon regeneration was evaluated by βIII‑tubulin immunostaining. Compared with recombinant human BDNF (rhBDNF) treatment as a positive control and EGFP OE as a negative control, Zdhhc17 OE significantly enhanced, whereas Zdhhc17 knockdown (KD) markedly impaired, axon regeneration (Figure 2C,D).

To further determine whether this phenotype depends on the PAT activity of Zdhhc17, we generated two enzymatic mutants: a catalytic domain‐deleted mutant (Zdhhc17ΔDHHC) [8] and an active‑site point mutant (Zdhhc17 C457S) [29] (Figure 2E,F and Figure S2A). In contrast to wild‑type (WT) Zdhhc17, both mutants lost the ability to promote axon regeneration after injury (Figure 2G,H). Additionally, the broad‐spectrum palmitoylation inhibitor 2‐bromopalmitate (2‑BP) exerted an inhibitory effect on axon regeneration in a dose‑dependent manner (Figure S2B). Collectively, Zdhhc17, acting as a PAT, effectively promotes axon regeneration in vitro.

### 3.3 Zdhhc17 Promotes Functional Recovery After SCI in a PAT‑Dependent Manner

**FIGURE 3:**
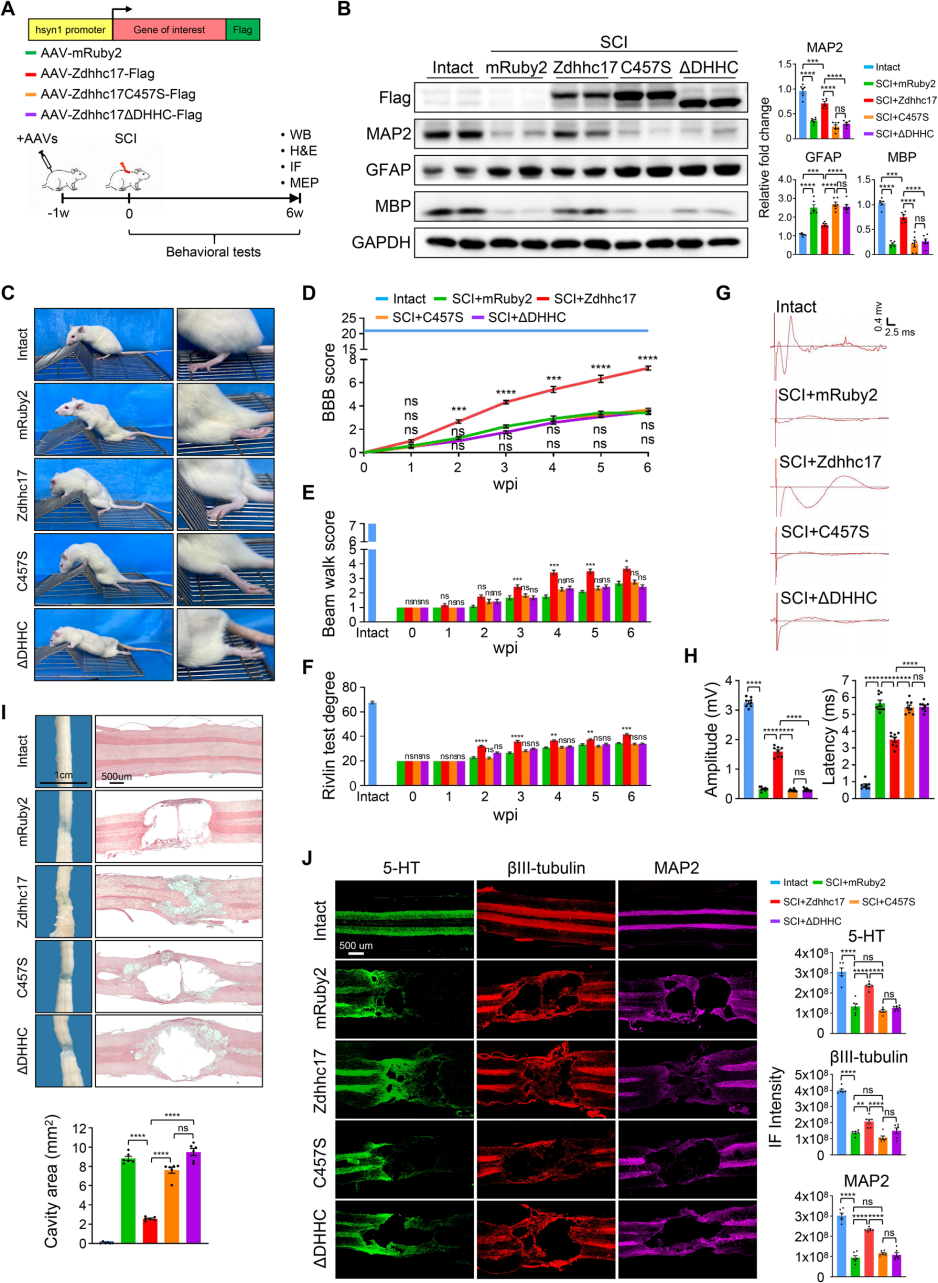
Zdhhc17 promotes functional recovery after SCI in a PAT‐dependent manner. (A) Schematic of the experimental design. (B) WB analysis of Flag‐tag, MAP2, GFAP, MBP, and GAPDH in the lesion sites from different groups. Right panels show quantification of the indicated protein expression levels. The color key is consistent across all panels. Data are presented as the mean ± SEM (*n* = 3 per group). ns, not significant; ^****^ *p* < 0.0001 by one‐way ANOVA. (C) Representative images of rats with different treatments at 6 wpi. (D–F) Behavioral assessments of functional recovery following SCI in groups with indicated treatments. Data are presented as mean ± SEM (*n* = 12/group/time point). ns, not significant; ^*^ *p* < 0.05; ^**^ *p* < 0.01; ^***^ *p* < 0.001; ^****^ *p* < 0.0001 by two‐way ANOVA comparing AAV‐mRuby2 with other groups. (G) Representative MEP recordings from rats with indicated treatments at 6 wpi. (H) Quantification of MEP amplitude and latency shown in (G). Data are presented as mean ± SEM (*n* = 6 per group). ns, not significant; ^****^ *p* < 0.0001 by one‐way ANOVA. (I) Representative images of the gross morphology of spinal cords at the T5‐L2 levels and H&E staining at the lesion sites. Lower panel, quantification of the lesion area. Data are presented as the mean ± SEM (*n* = 6 per group). ns, not significant; ^****^ *p* < 0.0001 by one‐way ANOVA. (J) Representative IF images of spinal cord coronal sections around injured site immunostained with antibodies against 5‐HT, βIII‐tubulin and MAP2 in rats with indicated treatments. Right panels show quantification of the indicated immunoreactive intensity. Data are presented as mean ± SEM (*n* = 6 per group). ns, not significant; ^**^ *p* < 0.01; ^****^ *p* < 0.0001 by one‐way ANOVA.

Based on the in vitro findings, we next asked whether the PAT activity of Zdhhc17 is required for its therapeutic effect on functional recovery after SCI in vivo (Figure 3A). Systematic structural and functional assessments showed that, under neuron‑specific expression, both enzymatic mutants, Zdhhc17ΔDHHC and Zdhhc17 C457S, completely failed to achieve beneficial outcomes after SCI, similar to the mRuby2 negative control (Figure 3B–J). To further assess locomotor performance in a non‑weight‑bearing context, swimming tests were performed at 4 wpi (Figure S3A). Intact rats displayed stable, hindlimb‑dominant swimming with coordinated limb movements (Figure S3B,C). By contrast, mRuby2 and Zdhhc17ΔDHHC rats showed rigid hindlimbs, severe imbalance, and a drooping tail, with propulsion dominated by forelimbs. Zdhhc17 rats, however, exhibited improved balance and more coordinated hindlimb movements, suggesting better motor recovery in the swimming task. The improved locomotor performance induced by Zdhhc17 implied reconstruction or remodeling of descending motor circuits rather than nonspecific neuronal preservation [30]. To test this, we performed BDA tracing to evaluate the effect of neuron‐specific Zdhhc17 restoration on CST axons by quantifying BDA‐labeled fibers (Figure S3D). In the intact group, BDA‐positive CST fibers were robustly detected at both T5 (rostral to the lesion) and T10 (the lesion site) levels (Figure S3E,F). At the T5 level, SCI caused significantly reduced BDA labeling in both mRuby2 and Zdhhc17ΔDHHC groups, reflecting secondary pathological changes after SCI, including retrograde degeneration, remote CST atrophy [31], impaired axonal transport [32], and persistent inflammatory responses [33]. At the T10 level, BDA signal was barely detected in either AAV‑mRuby2 or AAV‑Zdhhc17ΔDHHC group, indicating severe damage to descending corticospinal projections by SCI. Importantly, Zdhhc17 OE significantly restored these defects. Taken together, these results demonstrate that the therapeutic efficacy of Zdhhc17 in SCI repair strictly depends on its PAT activity.

### 3.4 Zdhhc17 Binds and Palmitoylates Kpna2, Kpnb1 and Ipo9

**FIGURE 4:**
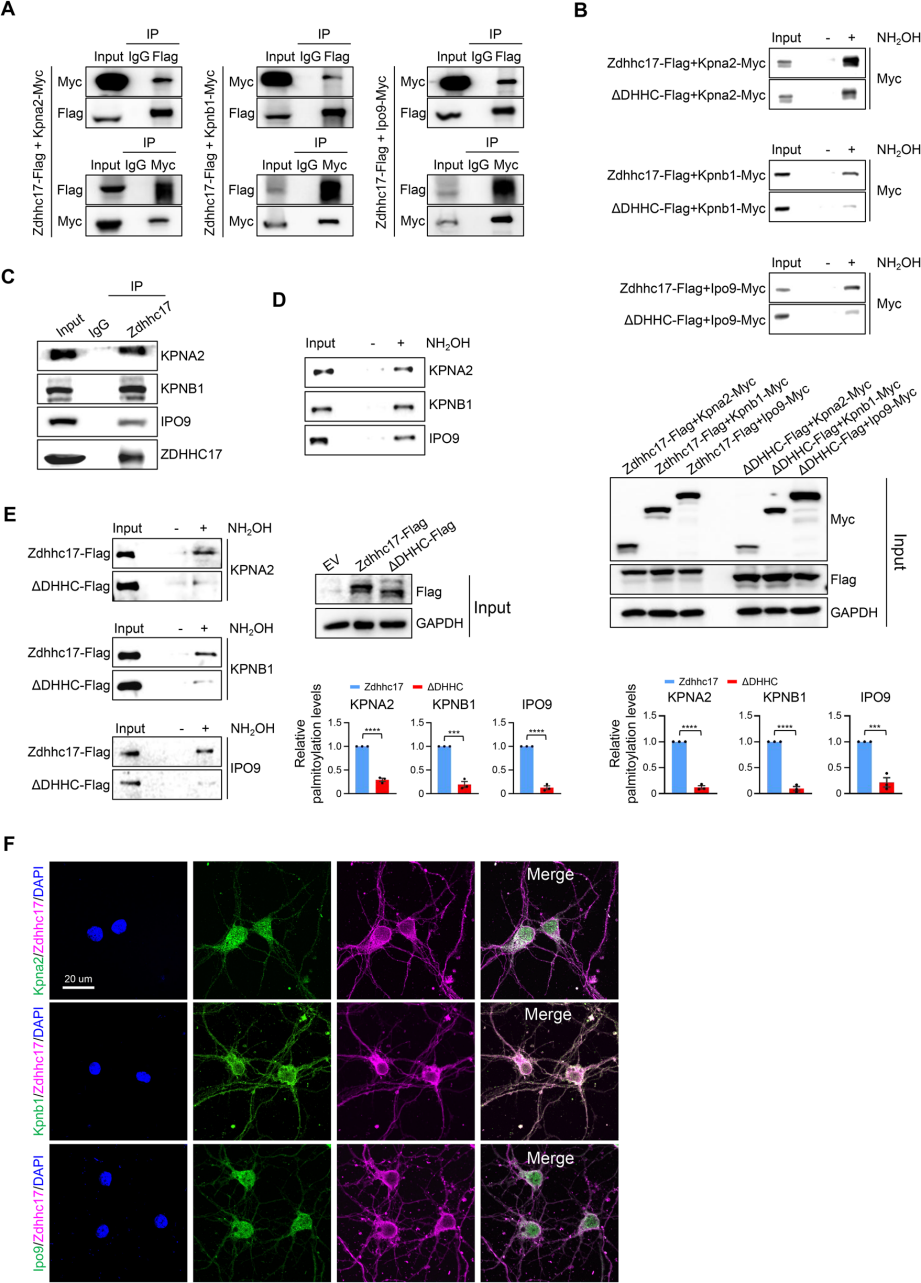
Zdhhc17 binds and palmitoylates Kpna2, Kpnb1 and Ipo9. (A) Reciprocal IP‐WB validation of the interactions between Zdhhc17 and the three Kaps in HEK293T cells. (B) Acyl‐RAC assay detecting palmitoylation of Kpna2, Kpnb1, and Ipo9 in HEK293T cells expressing WT or DHHC‐dead mutant of Zdhhc17. The bottom panels show quantification of the relative palmitoylation levels. Data are presented as the mean ± SEM (*n* = 3 per group). ^***^ *p* < 0.001 ^****^ *p* < 0.0001 by one‐way ANOVA. (C) IP‐WB validation of the endogenous interaction of Zdhhc17 with Kpna2, Kpnb1, and Ipo9 in primary neurons. (D) Acyl‐RAC assay detecting palmitoylation of the karyopherins in primary neurons. (E) Acyl‐RAC assay detecting palmitoylation of Kpna2, Kpnb1, and Ipo9 in primary neurons expressing WT or DHHC‐dead mutant of Zdhhc17. The bottom right panels show quantification of the relative palmitoylation levels. Data are presented as the mean ± SEM (*n* = 3 per group). ^***^ *p* < 0.001 ^****^ *p* < 0.0001 by one‐way ANOVA. (F) Representative IF images showing the colocalization of Zdhhc17 with Kpna2, Kpnb1, and Ipo9 in primary neurons.

To elucidate the molecular mechanisms by which Zdhhc17 promotes SCI repair, we first profiled the Zdhhc17 interactome in cortical neurons by immunoprecipitation followed by mass spectrometry (IP‑MS) (Figure S4A). Interestingly, Zdhhc17 co‑immunoprecipitated with nine members of the karyopherin family (Figure S4B). Among these, interactions with Kpna2, Kpnb1, and Ipo9 were confirmed by reciprocal co‑immunoprecipitation in co‐transfected HEK293T cells (Figure 4A).

To determine whether these Kaps are palmitoylated, we ectopically expressed Zdhhc17 WT‐Flag together with each of the nine Myc‐tagged Kaps in HEK293T cells and performed Acyl Resin Assisted Capture (Acyl‐RAC) assays. Kpna2, Kpnb1, and Ipo9 consistently showed clear palmitoylation signals (Figure 4B and Figure S4C). Moreover, Acyl‐RAC analysis of lysates from HEK293T cells co‐transfected with the three Kaps and Zdhhc17ΔDHHC revealed markedly reduced palmitoylation of all three, further supporting that Zdhhc17 palmitoylates Kpna2, Kpnb1, and Ipo9.

We next asked whether Zdhhc17 interacts with and palmitoylates endogenous Kpna2, Kpnb1, and Ipo9 in neurons. IP‑WB and IF staining in primary cortical neurons confirmed that Zdhhc17 co‐immunoprecipitated and colocalized with each of the three Kaps (Figure 4C,F). Moreover, Acyl‐RAC analysis revealed that endogenous Kpna2, Kpnb1, and Ipo9 were palmitoylated in neurons (Figure 4D), and that their palmitoylation was significantly reduced by Zdhhc17ΔDHHC compared with Zdhhc17 WT (Figure 4E). Taken together, these data identify Kpna2, Kpnb1, and Ipo9 as novel palmitoylation substrates of Zdhhc17 in neurons, which were selected for further investigation.

### 3.5 Zdhhc17‑Mediated Palmitoylation Stabilizes Kpna2 and Ipo9 Proteins by Suppressing Their Ubiquitin‑Dependent Degradation

**FIGURE 5:**
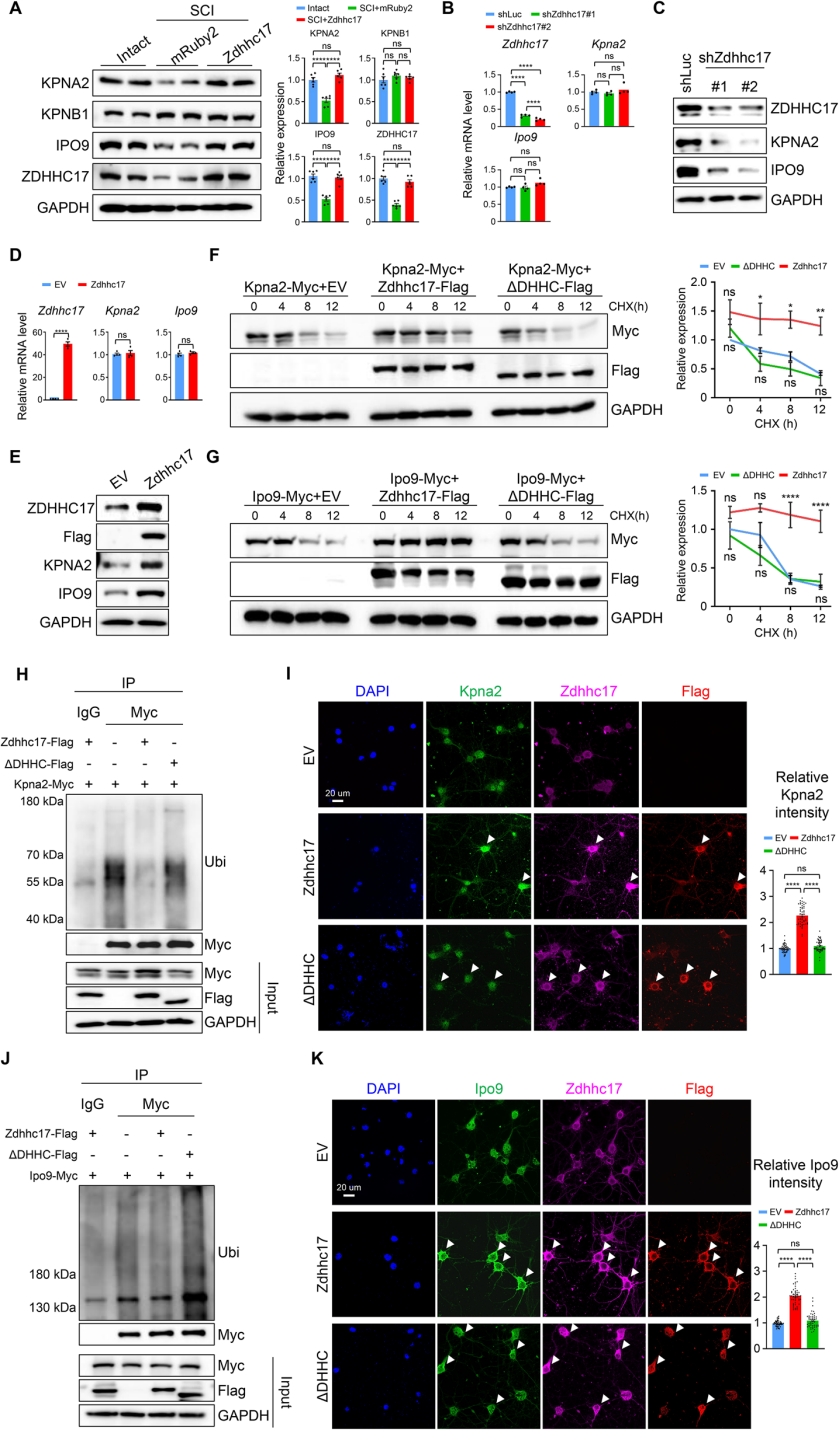
Zdhhc17‐mediated palmitoylation stabilizes KPNA2 and IPO9 by suppressing ubiquitin‐dependent degradation. (A) WB analysis of Kpna2, Kpnb1, and Ipo9 expression in spinal cord lysates from the lesion sites of SCI rats with or without neuron‐specific Zdhhc17 overexpression. Right panels show quantification of the indicated protein levels. Data are presented as the mean ± SEM (*n* = 3 per group). ns, not significant; ^****^ *p* < 0.0001 by one‐way ANOVA. (B) Real‐time PCR analysis of Kpna2 and Ipo9 mRNA levels in primary neurons following shRNA‐mediated Zdhhc17 knockdown. Data are presented as mean ± SEM (*n* = 4). ns, not significant; ^****^ *p* < 0.0001 by one‐way ANOVA. (C) WB analysis of Kpna2 and Ipo9 protein levels in primary neurons upon shRNA‐mediated Zdhhc17 knockdown. (D) Real‐time PCR analysis of Kpna2 and Ipo9 mRNA levels in primary neurons upon Zdhhc17 overexpression. Data are presented as mean ± SEM (*n* = 6). ns, not significant; ^****^ *p* < 0.0001 by one‐way ANOVA. (E) WB analysis of Kpna2 and Ipo9 protein levels in primary neurons upon Zdhhc17 overexpression. (F, G) WB analysis of Myc‑tagged Kpna2 (F) and Ipo9 (G) in HEK293T cells co‐expressing EV, Zdhhc17 WT, or Zdhhc17ΔDHHC, following a cycloheximide (CHX) time‐course treatment. Right panels show quantification of the indicated protein levels. Data are presented as the mean ± SEM (*n* = 3 per group). ns, not significant; ^*^ *p* < 0.05; ^**^ *p* < 0.01; ^****^ *p* < 0.0001 by two‐way ANOVA comparing EV with other groups. (H) Ubiquitination assay of KPNA2 in HEK293T cells expressing Zdhhc17 WT or Zdhhc17ΔDHHC following MG132 treatment. (I) Representative IF images of primary neurons expressing Flag‐tagged Zdhhc17 WT or Zdhhc17ΔDHHC, immunostained for Kpna2, Zdhhc17, and Flag. Triangles indicate representative Flag‑positive neurons. The right panel shows quantification of relative Kpna2 intensity per neuron. Data are presented as the mean ± SEM (*n* = 50 per group). ns, not significant; ^****^ *p* < 0.0001 by one‐way ANOVA. (J) Ubiquitination assay of IPO9 in HEK293T cells expressing Zdhhc17 WT or Zdhhc17ΔDHHC following MG132 treatment. (K) Representative IF images of primary neurons expressing Flag‐tagged Zdhhc17 WT or Zdhhc17ΔDHHC, immunostained for Ipo9, Zdhhc17, and Flag. Triangles indicate representative Flag‑positive neurons. The right panel shows quantification of relative Ipo9 intensity per neuron. Data are presented as the mean ± SEM (*n* = 50 per group). ns, not significant; ^****^ *p* < 0.0001 by one‐way ANOVA.

We next asked how Zdhhc17‑mediated palmitoylation regulates Kpna2, Kpnb1, and Ipo9. To assess the physiological impact of Zdhhc17 OE on these Kaps, we first evaluated their protein levels in spinal cord tissue from SCI rats with or without neuron‑specific Zdhhc17 OE. SCI significantly reduced Kpna2 and Ipo9 protein levels, whereas Zdhhc17 OE substantially restored them, indicating a positive correlation between Zdhhc17 expression and Kpna2/Ipo9 protein abundance (Figure 5A). Therefore, we prioritized these two Kaps for follow‑up and did not pursue Kpnb1. Consistent with the in vivo findings, in primary cortical neurons, Zdhhc17 KD decreased (Figure 5B,C), whereas Zdhhc17 OE increased (Figure 5D,E), Kpna2 and Ipo9 protein levels to a statistically significant degree, without affecting their mRNA levels. To investigate whether Zdhhc17 controls protein stability, we measured the half‑lives of Myc‑tagged Kpna2 and Ipo9 in HEK293T cells expressing empty vector (EV), Zdhhc17 WT, or the catalytic mutant Zdhhc17ΔDHHC, following cycloheximide (CHX) treatment. Zdhhc17 WT, but not Zdhhc17ΔDHHC, significantly increased the stability of both Kpna2 and Ipo9 (Figure 5F,G).

Given that Zdhhc17 has been reported to suppress substrate polyubiquitination and protein degradation [34], we tested whether a similar mechanism applies to Kpna2 and Ipo9. Upon MG132 treatment, ubiquitination assays showed that both KPNA2 and IPO9 exhibited reduced ubiquitination in the presence of Zdhhc17 WT, but not Zdhhc17ΔDHHC (Figure 5H,J), indicating that Zdhhc17 stabilizes these Kaps by preventing ubiquitin‑dependent degradation. Moreover, co‑IP assays showed that the Zdhhc17 mutants still interact with Kpna2 and Ipo9 (Figure S5A,B), excluding loss of binding as an explanation for the difference in their ubiquitination and stability.

We next asked whether Zdhhc17, acting as a PAT, influences the subcellular localization of Kpna2 and Ipo9 in primary cortical neurons. Immunofluorescence revealed that lentiviral expression of Flag‑Zdhhc17 WT, but not Zdhhc17ΔDHHC, significantly increased the protein abundance of both Kpna2 (Figure 5I) and Ipo9 (Figure 5K), consistent with our previous results. Kpna2 distribution was essentially unaffected by Zdhhc17 levels, remaining relatively even between the nucleus and soma. By contrast, Ipo9 localization was sensitive to Zdhhc17 expression: in neurons expressing EV or Zdhhc17ΔDHHC, Ipo9 was distributed relatively evenly between the nucleus and soma, whereas Zdhhc17 WT OE led to a noticeable accumulation of Ipo9 in the perinuclear region.

**FIGURE 6:**
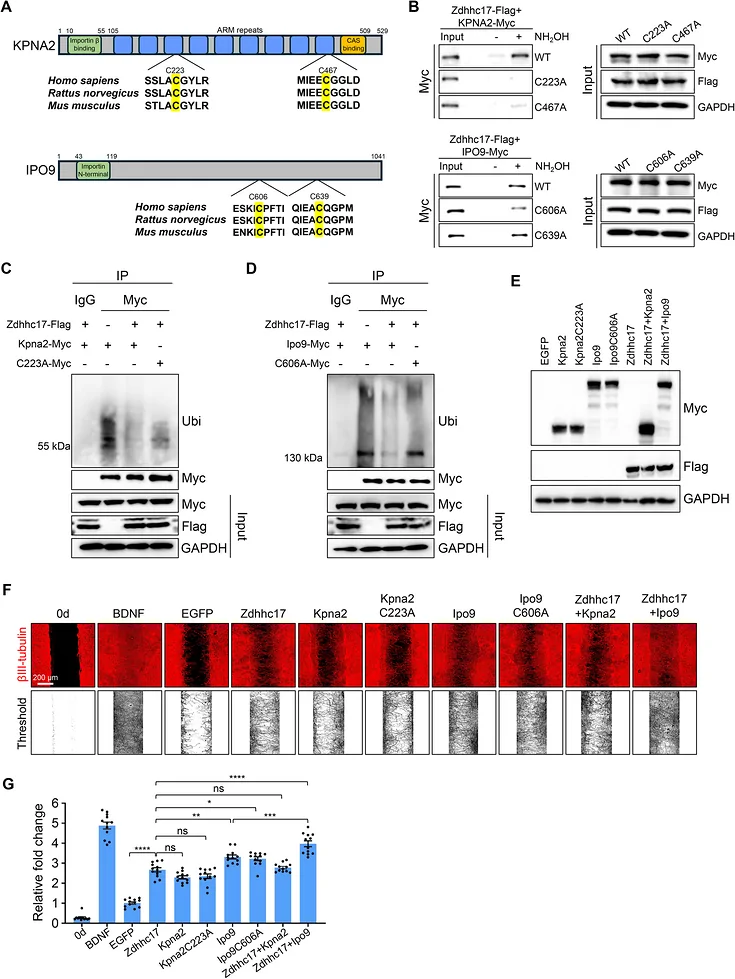
Palmitoylation of KPNA2 and IPO9 at Cys223 and Cys606 promotes their stability and axon regeneration. (A) Schematic of predicted palmitoylation sites in KPNA2 and IPO9. (B) Acyl‐RAC assay detecting palmitoylation of KPNA2 and IPO9 WT and cysteine‐to‐alanine mutants in HEK293T cells upon Zdhhc17 co‐expression. Right panels, input controls. (C) Ubiquitination assay of KPNA2 WT or KPNA2 C223A in HEK293T cells expressing Zdhhc17 following MG132 treatment. (D) Ubiquitination assay of IPO9 WT or IPO9 C606A in HEK293T cells expressing Zdhhc17 following MG132 treatment. (E) WB analysis of primary neurons individually overexpressing WT Kpna2 or Ipo9, their palmitoylation‑site mutants, and each combined with Zdhhc17. (F) Representative images of scratch‐injured primary neurons overexpressing WT Kpna2 or Ipo9, their palmitoylation‑site mutants, and each co‑expressed with Zdhhc17, immunostained for βIII‐tubulin, with corresponding ImageJ‑thresholded images. (G) Quantification of βIII‐tubulin intensity in the scratched area upon various treatments in (F). Data are presented as mean ± SEM (*n* = 12). ns, not significant; ^*^ *p* < 0.05, ^**^ *p* < 0.01, ^***^ *p* < 0.001, ^****^ *p* < 0.0001 by one‐way ANOVA.

To map putative Zdhhc17‐targeted sites, we used CSS‐Palm to predict conserved palmitoylation sites in KPNA2 and IPO9 (Figure 6A). For each protein, we selected two high‐confidence predicted sites and generated cysteine‐to‐alanine point mutants: KPNA2 C223A and C467A; IPO9 C606A and C639A. Palmitoylation of the KPNA2 C223A and IPO9 C606A mutants was extensively reduced compared with their WT counterparts, indicating that these cysteines are the primary Zdhhc17‑dependent palmitoylation sites (Figure 6B). To further examine whether these mutations affect ubiquitination‐mediated protein degradation, HEK293T cells overexpressing Zdhhc17 WT together with either WT or palmitoylation‐site mutants were treated with the proteasome inhibitor MG132. The palmitoylation‐site mutants showed increased ubiquitination levels for both Kaps, reinforcing that Zdhhc17 stabilizes KPNA2 and IPO9 proteins via palmitoylation (Figure 6C,D). To rule out impaired binding as the cause of reduced palmitoylation, we performed co‑IP and confirmed that ZDHHC17 still interacts with the KPNA2 C223A and IPO9 C606A mutants (Figure S5C,D). Thus, these data demonstrate that Zdhhc17 palmitoylates KPNA2 and IPO9 to enhance their protein stability.

To preliminarily test the functional roles of Kpna2 and Ipo9, we overexpressed their WT and mutant forms and employed the in vitro neuronal scratch model (Figure 6E). Both Kpna2 and Ipo9 significantly enhanced axon regeneration compared with EGFP (Figure 6F,G). Functionally, Kpna2 was less potent than Zdhhc17, whereas Ipo9 exerted a more robust pro‑regenerative effect than Zdhhc17. Notably, overexpression of the palmitoylation‑deficient mutants Kpna2 C223A and Ipo9 C606A promoted axon regeneration to a similar extent as their WT counterparts, suggesting that elevating Kap protein levels in injured neurons is a major mechanism by which Zdhhc17 facilitates axon regeneration. To explore potential synergy between Zdhhc17 and its substrates, we co‑expressed Zdhhc17 with Kpna2 or Ipo9 in neurons. The combination of Zdhhc17 and Ipo9 produced a greater pro‑regenerative effect than either protein alone, whereas co‑expression of Zdhhc17 and Kpna2 yielded an additional increase that did not reach statistical significance over Zdhhc17 alone (Figure 6F,G). Taken together, these findings indicate that Kpna2 and Ipo9 act as pro‑regenerative substrates of Zdhhc17, with Ipo9 displaying cooperative effects with Zdhhc17.

### 3.6 Ipo9 and Kpna2 Recapitulate Zdhhc17‑Mediated SCI Repair, and Ipo9 Cooperates With Zdhhc17 to Further Enhance Recovery

**FIGURE 7:**
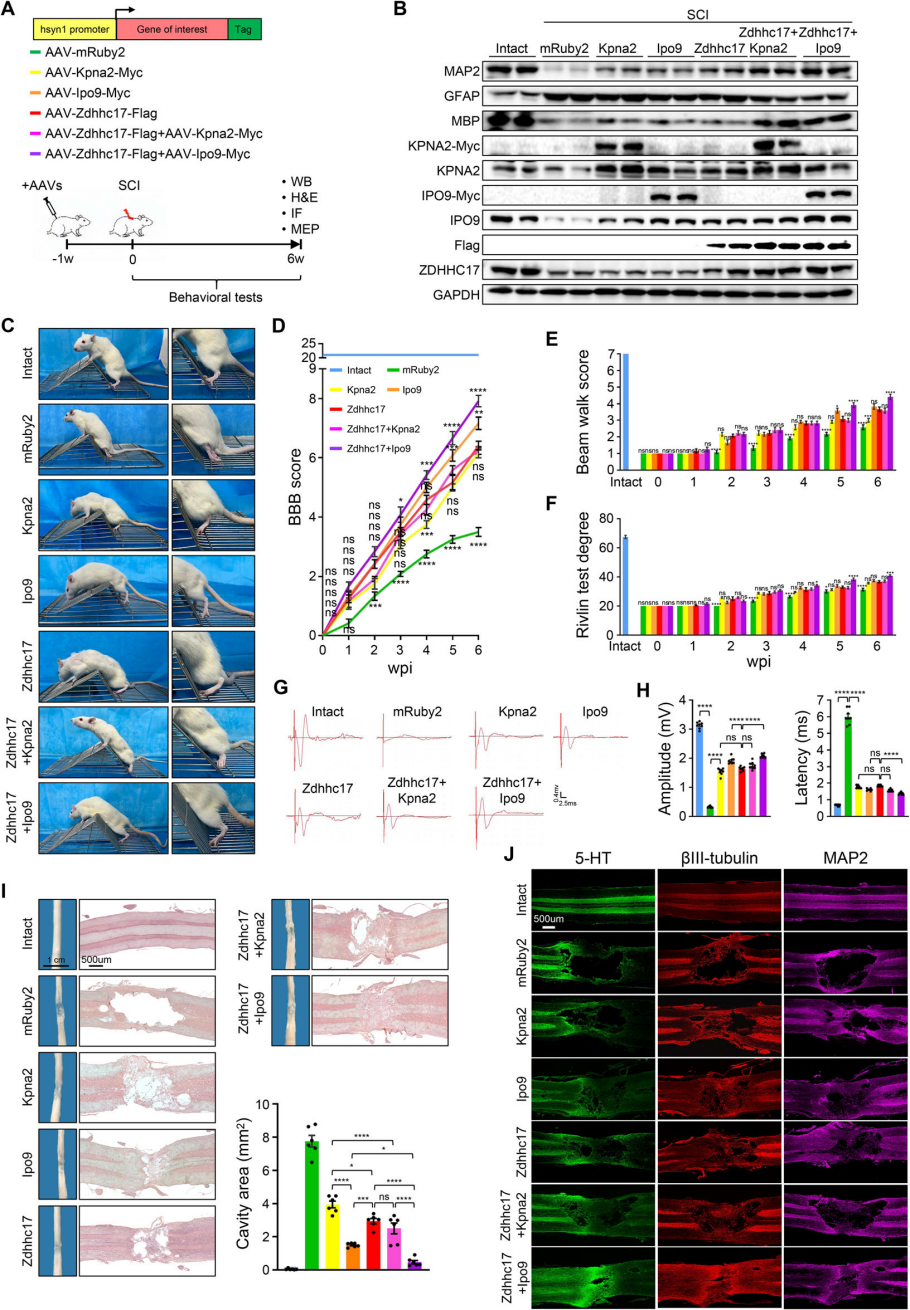
Ipo9 and Kpna2 recapitulate Zdhhc17‑mediated SCI repair, and Ipo9 cooperates with Zdhhc17 to further enhance recovery. (A) Schematic of the experimental design. (B) WB analysis of Flag, Myc, MAP2, GFAP, MBP, and GAPDH in the lesion sites from different groups. (C) Representative images of rats with different treatments at 6 wpi. (D–F) Behavioral assessments of functional recovery following SCI in groups with indicated treatments. Data are presented as mean ± SEM (*n* = 12/group/time point). ns, not significant; ^*^ *p* < 0.05; ^***^ *p* < 0.001; ^****^ *p* < 0.0001 by two‐way ANOVA comparing AAV‐Zdhhc17 with other groups. (G) Representative MEP recordings from rats with indicated treatments at 6 wpi. (H) Quantification of MEP amplitude and latency. Data are presented as mean ± SEM (*n* = 6 per group). ns, not significant; ^****^ *p* < 0.0001 by one‐way ANOVA. (I) Representative images of the gross morphology of spinal cords and H&E staining of spinal cords at the lesion sites for various treatments. Bottom‐right panel, quantification of the lesion area. Data are presented as the mean ± SEM (*n* = 6 per group). ns, not significant; ^*^ *p* < 0.05; ^**^ *p* < 0.01, ^****^ *p* < 0.0001 by one‐way ANOVA. (J) Representative IF images of spinal cord coronal sections around injured site immunostained with antibodies against 5‐HT, βIII‐tubulin and MAP2 in rats with indicated treatments at the end of the experiment.

To determine whether Kpna2 and Ipo9, as Zdhhc17 substrates, also promote SCI repair in vivo, we overexpressed Myc‑tagged Kpna2 or Ipo9 in rat spinal neurons and compared their effects with Zdhhc17 OE. Integrated analyses of behavioral performance, electrophysiology, and histology showed that both Kpna2 and Ipo9 reproduced the beneficial effects of Zdhhc17 on SCI repair, with Ipo9 producing even more pronounced improvement (Figure 7B–J and Figure S6).

Because Zdhhc17, Kpna2, and Ipo9 each improved hindlimb locomotor recovery after SCI, and Zdhhc17 may act through additional substrates or pathways, we next asked whether combined restoration of Zdhhc17 with Kpna2 (Zdhhc17+Kpna2) or Ipo9 (Zdhhc17+Ipo9) could further enhance repair (Figure 7A). Co‑expression of Zdhhc17 and Kpna2 did not significantly outperform Zdhhc17 alone, whereas combinatorial expression of Zdhhc17 and Ipo9 produced superior recovery relative to either factor alone (Figure 7B–J and Figure S6). Thus, consistent with our in vitro findings, Kpna2 and Ipo9 promote SCI repair in vivo, and Ipo9 in particular acts synergistically with Zdhhc17 to further enhance functional recovery after SCI.

### 3.7 Zdhhc17 Promotes Neuronal Survival and Activates Transcription of Pro‑Regenerative Genes After Injury

**FIGURE 8:**
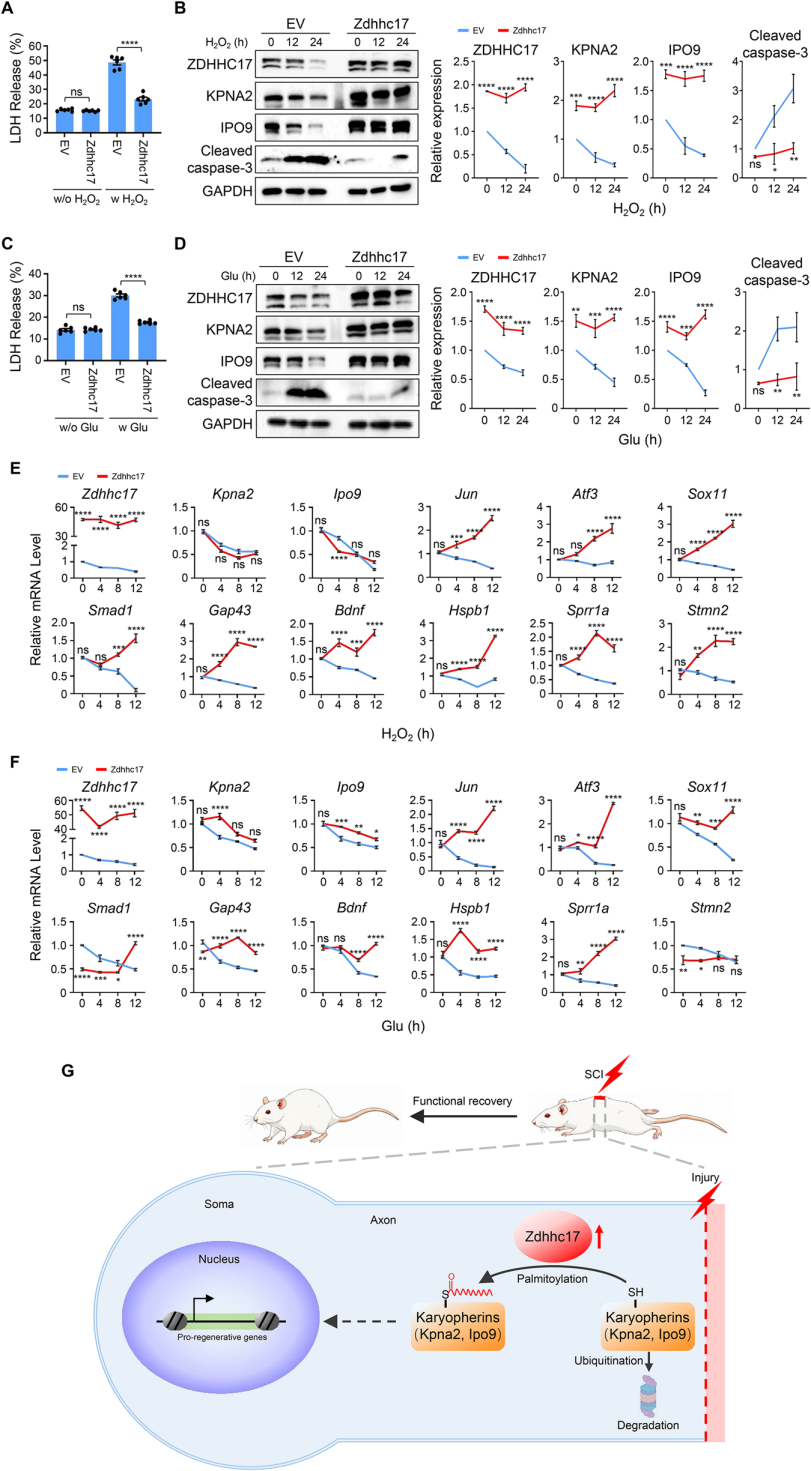
Zdhhc17 promotes neuronal survival and activates transcription of pro‑regenerative genes after injury. (A) Lactate dehydrogenase (LDH) release assay of primary cortical neurons overexpressing EV or Zdhhc17, with or without 24 h H_2_O_2_ treatment. Data are presented as mean ± SEM (*n* = 6 per group). ns, not significant; ^****^ *p* < 0.0001 by one‐way ANOVA. (B) WB analysis of cleaved caspase‐3, KPNA2, and IPO9 protein levels in primary neurons overexpressing EV or Zdhhc17 at 0, 12, and 24 h after H_2_O_2_ treatment. Right panels show quantification of the indicated protein expression levels. Data are presented as mean ± SEM (*n* = 3 per group). ns, not significant; ^*^ *p* < 0.05; ^**^ *p* < 0.01; ^***^ *p* < 0.001; ^****^ *p* < 0.0001 by two‐way ANOVA. (C) LDH release assay of primary cortical neurons overexpressing EV or Zdhhc17, with or without 24 h glutamate treatment. Data are presented as mean ± SEM (*n* = 6 per group). ns, not significant; ^****^ *p* < 0.0001 by one‐way ANOVA. (D) WB analysis of cleaved caspase‐3, KPNA2, and IPO9 protein levels in primary cortical neurons overexpressing EV or Zdhhc17 at 0, 12, and 24 h after glutamate treatment. Right panels show quantification of the indicated protein expression levels. Data are presented as mean ± SEM (*n* = 3 per group). ns, not significant; ^**^ *p* < 0.01; ^***^ *p* < 0.001; ^****^ *p* < 0.0001 by two‐way ANOVA. (E, F) Real‐time PCR analysis of Kpna2, Ipo9, c‐Jun, Atf3, Sox11, Smad1, Gap43, Bdnf, Hspb1, Sprr1a, and Stmn2 mRNA levels in primary cortical neurons overexpressing EV or Zdhhc17 following a time‑course H_2_O_2_ (E) or glutamate (F) treatment. Data are presented as mean ± SEM (*n* = 4). ns not significant; ^*^ *p* < 0.05; ^**^ *p* < 0.01; ^***^ *p* < 0.001; ^****^ *p* < 0.0001 by two‐way ANOVA. (G) Proposed model of the Zdhhc17‐Kpna2/Ipo9 axis in protecting CNS neurons after SCI. SCI reduces the expression of Zdhhc17, Kpna2, and Ipo9 in injured neurons. Restoration of Zdhhc17 palmitoylates Kpna2 and Ipo9, maintaining their protein levels by suppressing ubiquitin‑dependent degradation. Stabilized Kpna2/Ipo9 promote nuclear import of pro‑regenerative signals to activate intrinsic regeneration programs, leading to enhanced neuronal survival, axon regeneration, and improved functional recovery.

Oxidative stress and excitotoxicity are two well‑established major stresses faced by neurons after SCI [35]. To explore the detailed mechanisms by which Zdhhc17 protects neurons against SCI‑related insults, we used hydrogen peroxide (H_2_O_2_)–induced oxidative stress and glutamate‐induced excitotoxicity models in primary cortical neurons. Twenty‑four hours after H_2_O_2_ or glutamate treatment, the viability of neurons transduced with lentiviral Zdhhc17 was higher than that of neurons transduced with EV (Figure S7). Neuronal injury was quantitatively assessed by measuring lactate dehydrogenase (LDH) release into the culture medium at 24 h after treatment. Compared with the EV control group, Zdhhc17 OE significantly reduced neuronal death under both H_2_O_2_ (Figure 8A) and glutamate (Figure 8C) conditions. Western blot analysis showed that Zdhhc17 OE not only significantly decreased the apoptotic marker cleaved Caspase‑3, but also strongly attenuated the H_2_O_2_‑ and glutamate‑induced reductions in KPNA2 and IPO9 protein levels (Figure 8B,D). However, Zdhhc17 OE did not prevent the injury‑induced decreases in Kpna2 and Ipo9 mRNA levels (Figure 8E,F). These findings are consistent with our earlier results showing that Zdhhc17 increases KPNA2 and IPO9 protein abundance via post‑translational palmitoylation rather than transcriptional regulation.

We next examined the effects of Zdhhc17 on the time‑course expression of representative pro‑regenerative genes in both injury models, including *c‑Jun, Atf3*, *Sox11, Smad1, Gap43*, *Bdnf*, *Hspb1*, *Sprr1a*, and *Stmn2* [2, 36, 37]. In control neurons, prolonged exposure to either H_2_O_2_ (Figure 8E) or glutamate (Figure 8F) significantly suppressed the transcription of these genes as well as Zdhhc17. In contrast, Zdhhc17 OE robustly activated this gene set, with a more pronounced effect under H_2_O_2_ treatment. To further explore whether Kpna2 and Ipo9 are required for Zdhhc17‐mediated transcriptional activation of pro‐regenerative genes, we knocked down Kpna2 or Ipo9 in primary cortical neurons overexpressing Zdhhc17 prior to injury (Figure S8A). Protein levels of both Kpna2 and Ipo9 were reduced to less than 50% of control (Figure S8B,C). The results showed that the activation of most pro‑regenerative genes was significantly suppressed by Kpna2 or Ipo9 depletion upon H_2_O_2_ (Figure S8D,E) or glutamate (Figure S8F,G) treatment. Collectively, these findings indicate that Zdhhc17 restoration may protect injured neurons by broadly activating intrinsic transcriptional pro‑regenerative programs through Kpna2 and Ipo9.

## 4 Discussion

In this study, we demonstrate that Zdhhc17, acting as a PAT, suppresses neuronal apoptosis and promotes axon regeneration after injury, thereby enhancing functional recovery following SCI. In this context, Kpna2 and Ipo9 serve as novel palmitoylation substrates of Zdhhc17, whose palmitoylation prevents the injury‑induced degradation of their proteins and contributes to neuronal protection and SCI repair (Figure 8G).

To our knowledge, the only study on karyopherin palmitoylation is by Brownlee and Heald in Xenopus, showing that palmitoylated importin α (the human homolog of KPNA2) partitions to the plasma membrane to regulate cell size [38]. Interestingly, in our search for downstream targets of Zdhhc17, we found that a number of karyopherin family members were enriched in Zdhhc17 IPs. In addition to Kpna2, Kpnb1, and Ipo9, Kpna3, Kpna4, Kpnb2, Kpnb3, Xpo1, and Xpo2 all showed varying degrees of interaction with Zdhhc17 (Figure S4B). Given that our in vivo data have already demonstrated that Zdhhc17 promotes SCI repair in a PAT‑activity–dependent manner (Figure 3), we focused on Kpna2, Kpnb1, and Ipo9, which both reciprocally interact with Zdhhc17 and are palmitoylated by it. Therefore, it is possible that other Kaps may also contribute to SCI repair through protein–protein interaction with Zdhhc17 rather than as its palmitoylation substrates. We next found that SCI markedly reduced Kpna2 and Ipo9 protein levels in the spinal cord, whereas Zdhhc17 OE effectively restored them (Figure 5A). By contrast, total Kpnb1 protein levels in the spinal cord showed no obvious correlation with SCI or Zdhhc17 OE (Figure 5A). These findings suggest that, unlike Kpna2 and Ipo9, Kpnb1 protein levels are likely regulated by additional factors beyond SCI and Zdhhc17. The previously reported injury‑induced local translation of Kpnb1 in dorsal root ganglion (DRG) neurons by Hanz et al. may be one such factor [14, 39]. To better focus on Zdhhc17‑dependent regulation of karyopherin expression, we therefore selected Kpna2 and Ipo9 for subsequent mechanistic studies. Surprisingly, in primary cortical neurons, altering Zdhhc17 expression did not affect Kpna2 or Ipo9 mRNA levels. Taken together, these results support the notion that Zdhhc17 likely regulates Kpna2 and Ipo9 protein abundance via palmitoylation, and that this post‑translational control of nuclear transport proteins contributes to its therapeutic effects in SCI.

Functionally, however, Zdhhc17 and its two substrates do not contribute equally to repair. Under single‑gene overexpression conditions, Ipo9 is the most potent, Zdhhc17 is slightly less effective, and Kpna2 is clearly the weakest. When co‑expressed with Zdhhc17, only the Zdhhc17+Ipo9 combination shows a clearly superior synergistic effect. These results suggest that Kpna2‑dependent effects may already be largely saturated under Zdhhc17 OE and that Kpna2 is unlikely to be the major limiting component in this pathway, whereas Ipo9 represents a more critical, rate‑limiting downstream node that can be further amplified.

### 4.1 Limitations of the Study

Although we show that injury markedly reduces Kpna2 and Ipo9 mRNA and protein levels in CNS neurons both in vitro and in vivo, and that Zdhhc17 overexpression restores Kpna2 and Ipo9 proteins by inhibiting their ubiquitin‑dependent degradation, our study does not provide direct evidence that Zdhhc17 functions through palmitoylating Kpna2 and Ipo9. Overexpression of Kpna2 or Ipo9 alone phenocopies the therapeutic effects of Zdhhc17 after SCI (Figure 7), and knockdown of Kpna2 or Ipo9 attenuates Zdhhc17‐mediated activation of the pro‐regenerative program (Figure S8), but these observations only indicate that maintaining Kpna2 and Ipo9 protein levels is required for Zdhhc17‑mediated repair. Consistently, in the in vitro scratch assay, overexpression of palmitoylation‑deficient Kpna2 and Ipo9 mutants produced similar pro‑regenerative effects to their WT counterparts (Figure 6F,G). Thus, the most direct evidence that Kpna2 and Ipo9 function as essential Zdhhc17 substrates in SCI repair would come from knock‑in mice carrying palmitoylation‑site mutations in Kpna2 and Ipo9, in which neuron‑specific Zdhhc17 overexpression improves functional recovery after SCI to a much lesser extent. Alternatively, using neuron‑specific targeted degradation of Kpna2 and/or Ipo9—for example, degron‑based strategies [40] or proteolysis‑targeting chimera (PROTAC)–based approaches [41]—could also be used to test whether loss of these substrates at the protein level similarly abolishes the therapeutic effect of Zdhhc17. These substrate‑specific genetic and degradation approaches were beyond the scope of the present study and remain important goals for future work.

In addition, this study does not yet define the molecular mechanisms by which overexpression of Zdhhc17 activates the transcription of pro‑regenerative genes after neuronal injury. Previous studies have shown that upregulation of Kpna2/Kpnb1 and the nuclear import of regeneration‑associated transcription factors are key steps in initiating regenerative programs [15, 42, 43]. By contrast, Ipo9 has been characterized as a molecular chaperone for histone nuclear import, but its function in neurons remains unknown [44, 45]. We therefore plan to perform systematic transcriptomic analyses of Zdhhc17, Kpna2, and Ipo9 gain‑of‑function in injured cortical neurons to elucidate, at a genome‑wide level, how they collectively activate the regulatory networks underlying central neuronal regeneration.

## Author Contributions

M. L. conceived and designed the experiments and wrote the manuscript. M. C. planned the experiments, conducted experiments and performed data analysis. M. C., H. C., X. Z., and H. L. conducted BDA tracing and swimming tests. S. D. performed molecular and cellular experiments. Y. W. provided hUC‐MSCs and performed animal experiments. F. L. generated viral vectors and performed animal experiments. R. T. performed data curation and animal experiments. P. L. provided hUC‐MSCs. L. H., B. L., and L. R. provided experimental platform support. All authors read and approved the final manuscript.

## Conflicts of Interest

The authors declare no conflicts of interest.

## Supporting information




**Supporting File 1**: advs77724‐sup‐0001‐SuppMat.pdf.

**Supporting File 2**: advs77724‐sup‐0002‐Figures.pdf.

## Data Availability

The data that support the findings of this study are available from the corresponding author upon reasonable request.

## References

[1] J. M. Griffin and F. Bradke , “Therapeutic Repair for Spinal Cord Injury: Combinatory Approaches to Address a Multifaceted Problem,” EMBO Molecular Medicine 12, no. 3 (2020): 11505, 10.15252/emmm.201911505. PMC7059014 32090481

[2] M. Mahar and V. Cavalli , “Intrinsic Mechanisms of Neuronal Axon Regeneration,” Nature Reviews Neuroscience 19, no. 6 (2018): 323–337, 10.1038/s41583-018-0001-8. 29666508 PMC5987780

[3] B. Zheng and M. H. Tuszynski , “Regulation of Axonal Regeneration After Mammalian Spinal Cord Injury,” Nature Reviews Molecular Cell Biology 24, no. 6 (2023): 396–413, 10.1038/s41580-022-00562-y. 36604586

[4] S. M. F , L. Abrami , M. E. Linder , S. X. Bamji , B. C. Dickinson , and F. G. van der Goot , “Mechanisms and Functions of Protein S‐acylation,” Nature Reviews Molecular Cell Biology 25 (2024): 488–509, 10.1038/s41580-024-00700-8. 38355760 PMC12010433

[5] M. S. Rana , P. Kumar , C. J. Lee , R. Verardi , K. R. Rajashankar , and A. Banerjee , “Fatty Acyl Recognition and Transfer by an Integral Membrane S‐acyltransferase,” Science 359, no. 6372 (2018): aao6326, 10.1126/science.aao6326. PMC6317078 29326245

[6] R. R. Singaraja , “HIP14, a Novel Ankyrin Domain‐containing Protein, Links Huntingtin to Intracellular Trafficking and Endocytosis,” Human Molecular Genetics 11, no. 23 (2002): 2815–2828, 10.1093/hmg/11.23.2815. 12393793

[7] A. Yanai , K. Huang , R. Kang , et al., “Palmitoylation of Huntingtin by HIP14 Is Essential for Its Trafficking and Function,” Nature Neuroscience 9, no. 6 (2006): 824–831, 10.1038/nn1702. 16699508 PMC2279235

[8] W. Shi , F. Wang , M. Gao , et al., “ZDHHC17 Promotes Axon Outgrowth by Regulating TrkA–Tubulin Complex Formation,” Molecular and Cellular Neuroscience 68 (2015): 194–202, 10.1016/j.mcn.2015.07.005. 26232532

[9] A. A. Petropavlovskiy , J. A. Kogut , A. Leekha , C. A. Townsend , and S. S. Sanders , “A Sticky Situation: Regulation and Function of Protein Palmitoylation With a Spotlight on the Axon and Axon Initial Segment,” Neuronal Signaling 5, no. 4 (2021): Ns20210005, 10.1042/ns20210005. 34659801 PMC8495546

[10] Y. Zhang , S. Fan , L. He , and L. Li , “The ZDHHC13/ZDHHC17 Subfamily: From Biological Functions to Therapeutic Targets of Diseases,” Pharmacological Research 209 (2024): 107418, 10.1016/j.phrs.2024.107418. 39306022

[11] G. Yang and M. S. Cynader , “Palmitoyl Acyltransferase zD17 Mediates Neuronal Responses in Acute Ischemic Brain Injury by Regulating JNK Activation in a Signaling Module,” The Journal of Neuroscience 31, no. 33 (2011): 11980–11991, 10.1523/jneurosci.2510-11.2011. 21849558 PMC6623198

[12] J. Niu , S. S. Sanders , H.‐K. Jeong , et al., “Coupled Control of Distal Axon Integrity and Somal Responses to Axonal Damage by the Palmitoyl Acyltransferase ZDHHC17,” Cell Reports 33, no. 7 (2020): 108365, 10.1016/j.celrep.2020.108365. 33207199 PMC7803378

[13] C. E. Wing , H. Y. J. Fung , and Y. M. Chook , “Karyopherin‐mediated Nucleocytoplasmic Transport,” Nature Reviews Molecular Cell Biology 23, no. 5 (2022): 307–328, 10.1038/s41580-021-00446-7. 35058649 PMC10101760

[14] S. Hanz , E. Perlson , D. Willis , et al., “Axoplasmic Importins Enable Retrograde Injury Signaling in Lesioned Nerve,” Neuron 40, no. 6 (2003): 1095–1104, 10.1016/s0896-6273(03)00770-0. 14687545

[15] K. Ben‐Yaakov , S. Y. Dagan , Y. Segal‐Ruder , et al., “Axonal Transcription Factors Signal Retrogradely in Lesioned Peripheral Nerve,” The EMBO Journal 31, no. 6 (2012): 1350–1363, 10.1038/emboj.2011.494. 22246183 PMC3321171

[16] R. B.‐T. Perry , E. Doron‐Mandel , E. Iavnilovitch , et al., “Subcellular Knockout of Importin β1 Perturbs Axonal Retrograde Signaling,” Neuron 75, no. 2 (2012): 294–305, 10.1016/j.neuron.2012.05.033. 22841314 PMC3408616

[17] D. Yudin , S. Hanz , S. Yoo , et al., “Localized Regulation of Axonal RanGTPase Controls Retrograde Injury Signaling in Peripheral Nerve,” Neuron 59, no. 2 (2008): 241–252, 10.1016/j.neuron.2008.05.029. 18667152 PMC2538677

[18] R. Ohara , Y. Fujita , K. Hata , M. Nakagawa , and T. Yamashita , “Axotomy Induces Axonogenesis in Hippocampal Neurons Through STAT3,” Cell Death & Disease 2, no. 6 (2011): 175, 10.1038/cddis.2011.59. PMC3169001 21697950

[19] G. K. Pathak , H. Ornstein , H. Aranda‐Espinoza , A. J. Karlsson , and S. B. Shah , “Increases in Retrograde Injury Signaling Complex‐Related Transcripts in Central Axons Following Injury,” Neural Plasticity 2016 (2016): 1–13, 10.1155/2016/3572506. PMC5099454 27847648

[20] M. Pacifici and F. Peruzzi , “Isolation and Culture of Rat Embryonic Neural Cells: A Quick Protocol,” Journal of Visualized Experiments: JoVE 63 (2012): 3965, 10.3791/3965. PMC3466945 22664838

[21] M. Li , H. Gou , B. K. Tripathi , et al., “An Apela RNA‐Containing Negative Feedback Loop Regulates p53‐Mediated Apoptosis in Embryonic Stem Cells,” Cell Stem Cell 16, no. 6 (2015): 669–683, 10.1016/j.stem.2015.04.002. 25936916 PMC4458197

[22] R. C. Challis , S. Ravindra Kumar , K. Y. Chan , et al., “Systemic AAV Vectors for Widespread and Targeted Gene Delivery in Rodents,” Nature Protocols 14, no. 2 (2019): 379–414, 10.1038/s41596-018-0097-3. 30626963 PMC13333184

[23] T. Cao , H. Chen , W. Huang , et al., “hUC‐MSC‐Mediated Recovery of Subacute Spinal Cord Injury Through Enhancing the Pivotal Subunits β3 and γ2 of the GABA A Receptor,” Theranostics 12, no. 7 (2022): 3057–3078, 10.7150/thno.72015. 35547766 PMC9065192

[24] N. Xu , E. Åkesson , L. Holmberg , and E. Sundström , “A Sensitive and Reliable Test Instrument to Assess Swimming in Rats With Spinal Cord Injury,” Behavioural Brain Research 291 (2015): 172–183, 10.1016/j.bbr.2015.05.004. 25986406

[25] T. T. Cao , H. Chen , M. Pang , et al., “Dose Optimization of Intrathecal Administration of human Umbilical Cord Mesenchymal Stem Cells for the Treatment of Subacute Incomplete Spinal Cord Injury,” Neural Regen Res 17 (2022): 1785–1794, 10.4103/1673-5374.332151. 35017439 PMC8820722

[26] E. L. Gorenberg , S. Massaro Tieze , B. Yücel , et al., “Identification of Substrates of Palmitoyl Protein Thioesterase 1 Highlights Roles of Depalmitoylation in Disulfide Bond Formation and Synaptic Function,” PLoS Biology 20, no. 3 (2022): 3001590, 10.1371/journal.pbio.3001590. PMC9004782 35358180

[27] E. Coombes , J. Jiang , X. P. Chu , et al., “Pathophysiologically Relevant Levels of Hydrogen Peroxide Induce Glutamate‐independent Neurodegeneration That Involves Activation of Transient Receptor Potential Melastatin 7 Channels,” Antioxidants & Redox Signaling 14, no. 10 (2011): 1815–1827, 10.1089/ars.2010.3549. 20812867 PMC3078500

[28] S. K. Kandy , M. M. Nimonkar , S. S. Dash , B. Mehta , and Y. S. Markandeya , “Astaxanthin Protection Against Neuronal Excitotoxicity via Glutamate Receptor Inhibition and Improvement of Mitochondrial Function,” Marine Drugs 20, no. 10 (2022): 645, 10.3390/md20100645. 36286468 PMC9605357

[29] S. Milde and M. P. Coleman , “Identification of Palmitoyltransferase and Thioesterase Enzymes That Control the Subcellular Localization of Axon Survival Factor Nicotinamide Mononucleotide Adenylyltransferase 2 (NMNAT2),” Journal of Biological Chemistry 289, no. 47 (2014): 32858–32870, 10.1074/jbc.M114.582338. 25271157 PMC4239634

[30] R. Li , H. Xing , Y. Shen , et al., “Pharmacological Microglial Inhibition Remodels the Scar Microenvironment to Support Reticulospinal Circuit Reconstruction after Spinal Cord Injury,” Advanced Science 13 (2026): 03966, 10.1002/advs.202503966. PMC12767085 41103249

[31] P. Freund , N. Weiskopf , N. S. Ward , et al., “Disability, Atrophy and Cortical Reorganization Following Spinal Cord Injury,” Brain 134, no. 6 (2011): 1610–1622, 10.1093/brain/awr093. 21586596 PMC3102242

[32] N. Kawakami , “In Vivo Imaging in Autoimmune Diseases in the Central Nervous System,” Allergology International 65, no. 3 (2016): 235–242, 10.1016/j.alit.2016.02.001. 26935215

[33] A. P. Tran , P. M. Warren , and J. Silver , “The Biology of Regeneration Failure and Success after Spinal Cord Injury,” Physiological Reviews 98, no. 2 (2018): 881–917, 10.1152/physrev.00017.2017. 29513146 PMC5966716

[34] O. Voytyuk , Y. Ohata , A. Moustakas , P. Ten Dijke , and C. H. Heldin , “Smad7 Palmitoylation by the S‐Acyltransferase zDHHC17 Enhances Its Inhibitory Effect on TGF‐β/Smad Signaling,” Journal of Biological Chemistry 300, no. 7 (2024): 107462, 10.1016/j.jbc.2024.107462. 38876303 PMC11277750

[35] X. Hu , W. Xu , Y. Ren , et al., “Spinal Cord Injury: Molecular Mechanisms and Therapeutic Interventions,” Signal Transduction and Targeted Therapy 8, no. 1 (2023): 245, 10.1038/s41392-023-01477-6. 37357239 PMC10291001

[36] Z. He and Y. Jin , “Intrinsic Control of Axon Regeneration,” Neuron 90, no. 3 (2016): 437–451, 10.1016/j.neuron.2016.04.022. 27151637

[37] V. Chandran , G. Coppola , H. Nawabi , et al., “A Systems‐Level Analysis of the Peripheral Nerve Intrinsic Axonal Growth Program,” Neuron 89, no. 5 (2016): 956–970, 10.1016/j.neuron.2016.01.034. 26898779 PMC4790095

[38] C. Brownlee and R. Heald , “Importin α Partitioning to the Plasma Membrane Regulates Intracellular Scaling,” Cell 176, no. 4 (2019): 805–815.e8, 10.1016/j.cell.2018.12.001. 30639102 PMC6368448

[39] S. Maday , A. E. Twelvetrees , A. J. Moughamian , and E. L. Holzbaur , “Axonal Transport: Cargo‐specific Mechanisms of Motility and Regulation,” Neuron 84, no. 2 (2014): 292–309, 10.1016/j.neuron.2014.10.019. 25374356 PMC4269290

[40] Z. Zhang , E. L. Mena , R. T. Timms , I. Koren , and S. J. Elledge , “Degrons: Defining the Rules of Protein Degradation,” Nature Reviews Molecular Cell Biology 26, no. 11 (2025): 868–883, 10.1038/s41580-025-00870-z. 40659789

[41] J. Cai , C. Chen , J. Wang , et al., “PROTAC: A Revolutionary Technology Propelling Small Molecule Drugs Into the Next Golden Age,” Frontiers in Oncology 15 (2025): 1676414, 10.3389/fonc.2025.1676414. 41220927 PMC12597787

[42] H. R. Katz , A. A. Arcese , O. Bloom , and J. R. Morgan , “Activating Transcription Factor 3 (ATF3) Is a Highly Conserved Pro‐Regenerative Transcription Factor in the Vertebrate Nervous System,” Frontiers in Cell and Developmental Biology 10 (2022): 824036, 10.3389/fcell.2022.824036. 35350379 PMC8957905

[43] J. K. Lerch , Y. R. Martínez‐Ondaro , J. L. Bixby , and V. P. Lemmon , “cJun Promotes CNS Axon Growth,” Molecular and Cellular Neuroscience 59 (2014): 97–105, 10.1016/j.mcn.2014.02.002. 24521823 PMC4008678

[44] A. Padavannil , P. Sarkar , S. J. Kim , et al., “Importin‐9 Wraps Around the H2A‐H2B Core to Act as Nuclear Importer and Histone Chaperone,” eLife 8 (2019): 43630, 10.7554/eLife.43630. PMC6453568 30855230

[45] V. Palacios , G. C. Kimble , T. L. Tootle , and M. Buszczak , “Importin‐9 Regulates Chromosome Segregation and Packaging in Drosophila Germ Cells,” Journal of Cell Science 134, no. 7 (2021): jcs258391, 10.1242/jcs.258391. 33632744 PMC8077489

