# Supplementary material for: Palmitoyl Acyltransferase Zdhhc17 Promotes Functional Recovery After Spinal Cord Injury by Targeting the Nuclear Transport Factors Kpna2 and Ipo9
Source: Adv Sci (Weinh). 2026 Sep 11:e77724. Online ahead of print. doi: 10.1002/advs.77724 (PMC13564187; doi:10.1002/advs.77724)
Supplement: Supplementary file 1 [file ADVS-9999-e77724-s001.pdf]

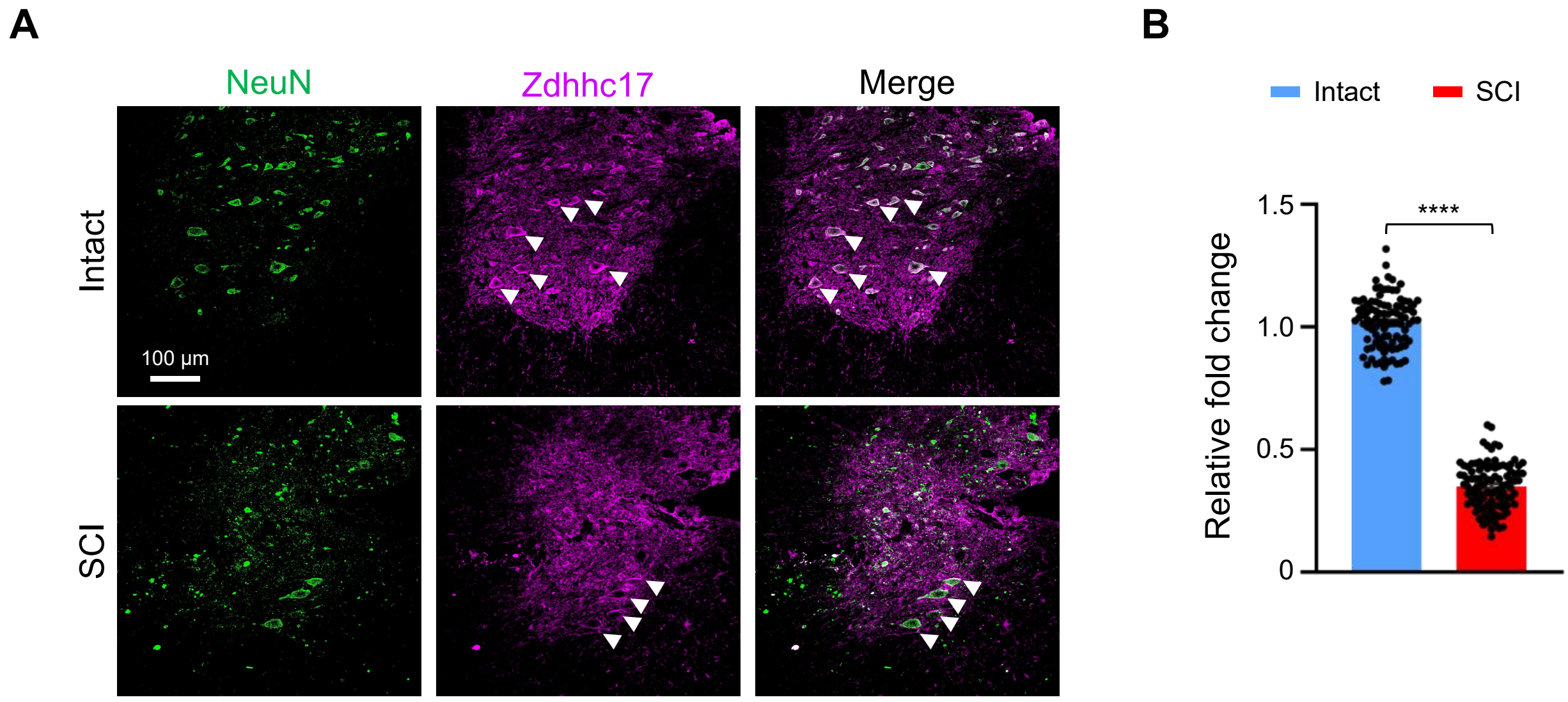
A
NeuN
Zdhhc17
Merge
Intact
SCI
100 μm
B
Intact
SCI
Relative fold change
1.5
1.0
0.5
0
****

Figure S1

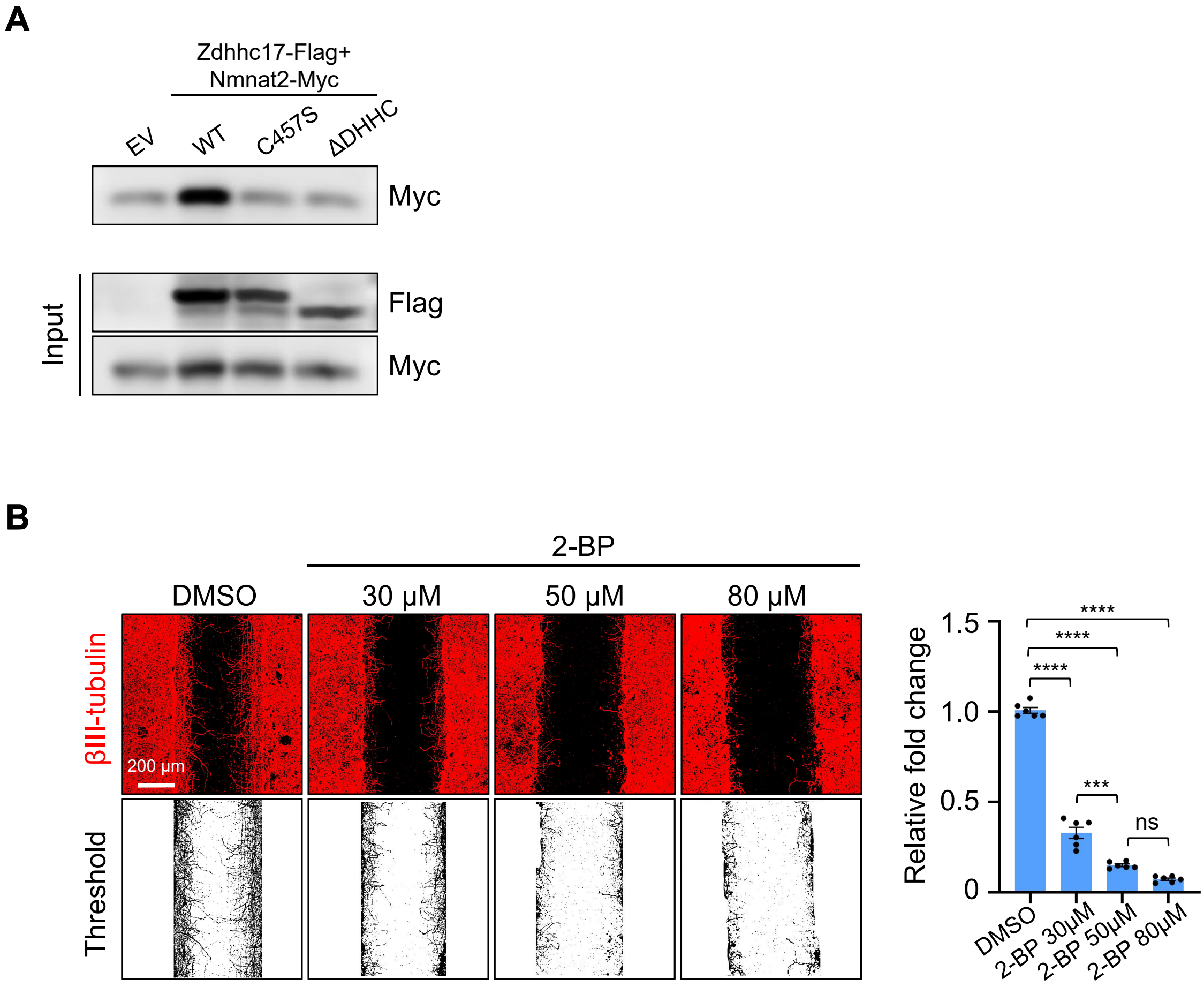
A
Zdhhc17-Flag+
Nmnat2-Myc
EV
WT
C457S
ΔDHHC
Myc
Input
Flag
Myc
B
2-BP
DMSO
30 μM
50 μM
80 μM
βIII-tubulin
200 μm
Threshold
Relative fold change
1.5
1.0
0.5
0
****
****
****
***
ns
DMSO
2-BP 30μM
2-BP 50μM
2-BP 80μM

Figure S2

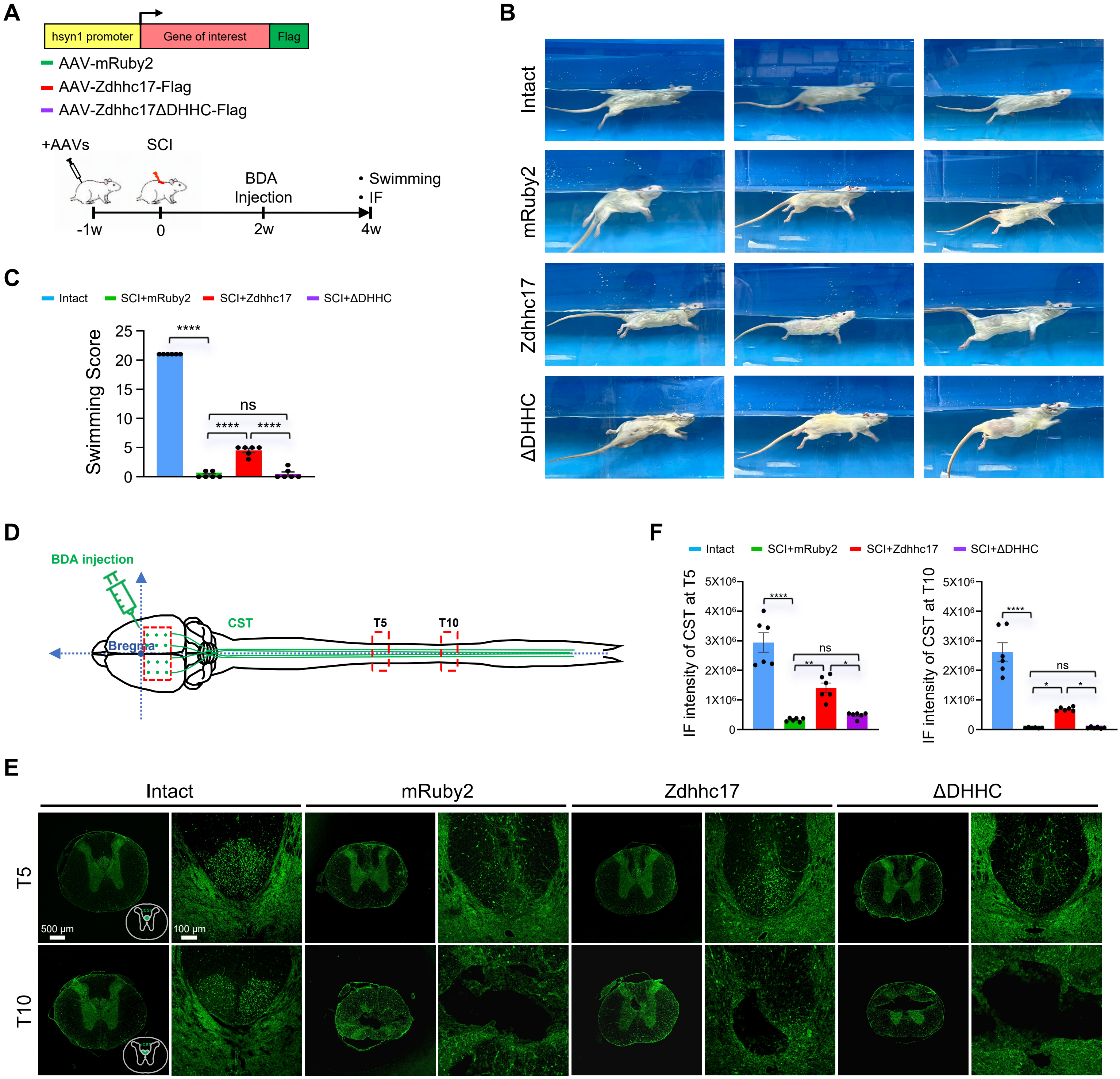
A
hsyn1 promoter
Gene of interest
Flag
AAV-mRuby2
AAV-Zdhhc17-Flag
AAV-Zdhhc17ΔDHHC-Flag
+AAVs
SCI
BDA
Injection
• Swimming
• IF
-1w
0
2w
4w
B
Intact
mRuby2
Zdhhc17
ΔDHHC
C
Intact
SCI+mRuby2
SCI+Zdhhc17
SCI+ΔDHHC
Swimming Score
ns
D
BDA injection
Bregma
CST
T5
T10
F
IF intensity of CST at T5
IF intensity of CST at T10
E
Intact
mRuby2
Zdhhc17
ΔDHHC
500 μm
100 μm

Figure S3

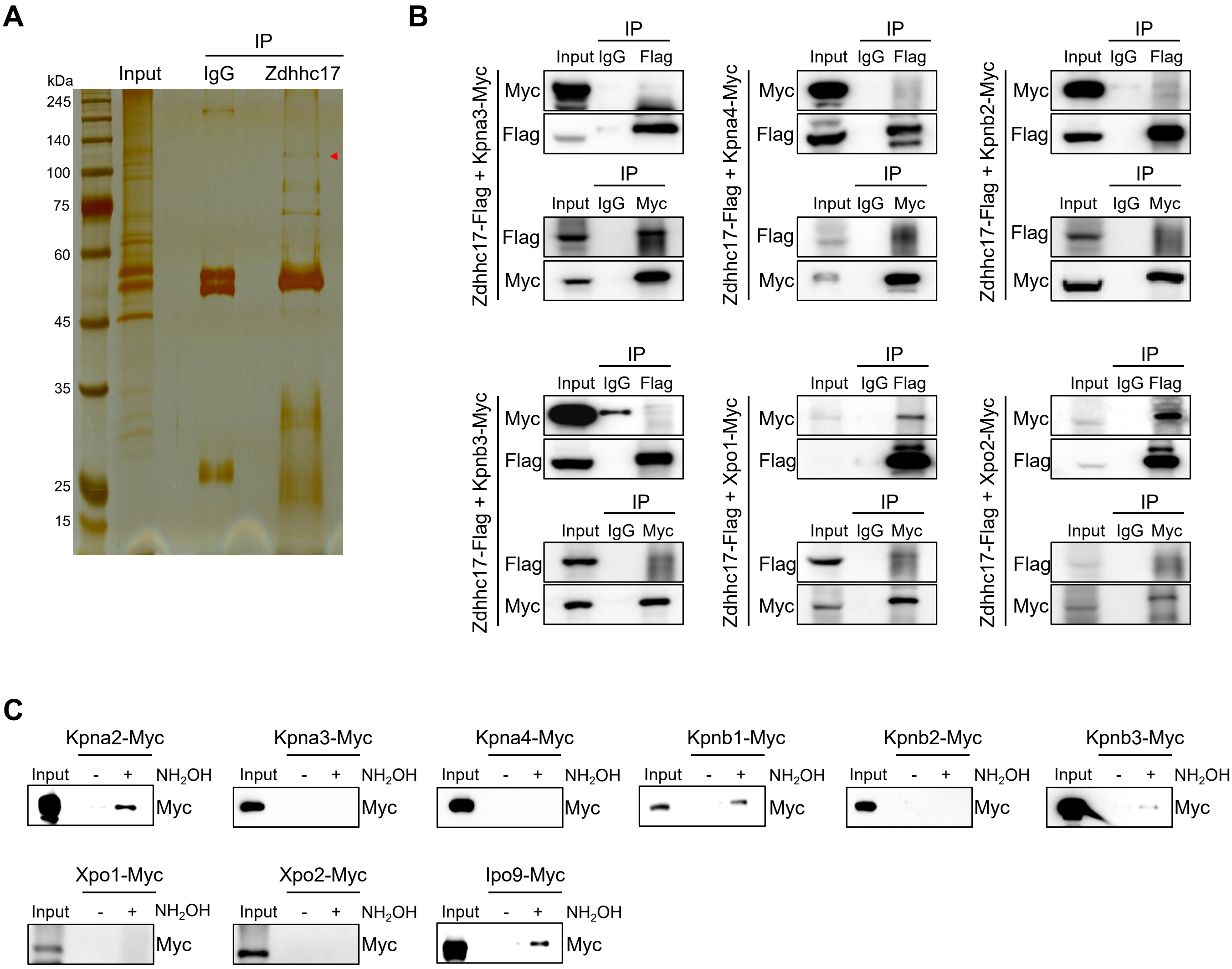
A
kDa
245
140
100
75
60
45
35
25
15
Input
IP
IgG
Zdhhc17
B
Zdhhc17-Flag + Kpna3-Myc
Zdhhc17-Flag + Kpna4-Myc
Zdhhc17-Flag + Kpnb2-Myc
Zdhhc17-Flag + Kpnb3-Myc
Zdhhc17-Flag + Xpo1-Myc
Zdhhc17-Flag + Xpo2-Myc
IP
Input IgG Flag
Input IgG Myc
Myc
Flag
C
Kpna2-Myc
Kpna3-Myc
Kpna4-Myc
Kpnb1-Myc
Kpnb2-Myc
Kpnb3-Myc
Xpo1-Myc
Xpo2-Myc
Ipo9-Myc
Input - + NH_2OH
Myc

Figure S4

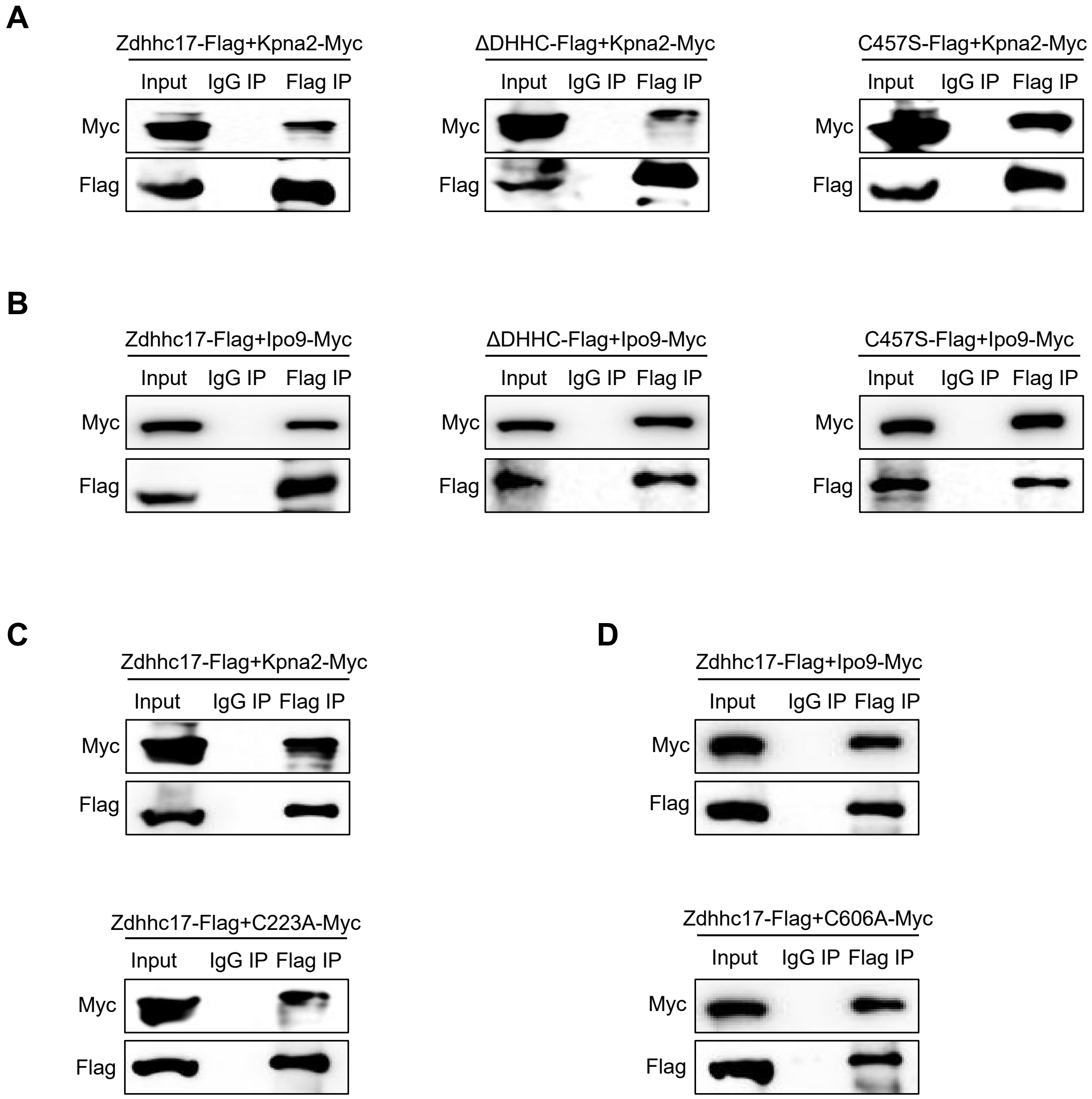
A
Zdhhc17-Flag+Kpna2-Myc
ΔDHHC-Flag+Kpna2-Myc
C457S-Flag+Kpna2-Myc
Input
IgG IP
Flag IP
Myc
Flag
B
Zdhhc17-Flag+Ipo9-Myc
ΔDHHC-Flag+Ipo9-Myc
C457S-Flag+Ipo9-Myc
C
Zdhhc17-Flag+Kpna2-Myc
Zdhhc17-Flag+C223A-Myc
D
Zdhhc17-Flag+Ipo9-Myc
Zdhhc17-Flag+C606A-Myc

Figure S5

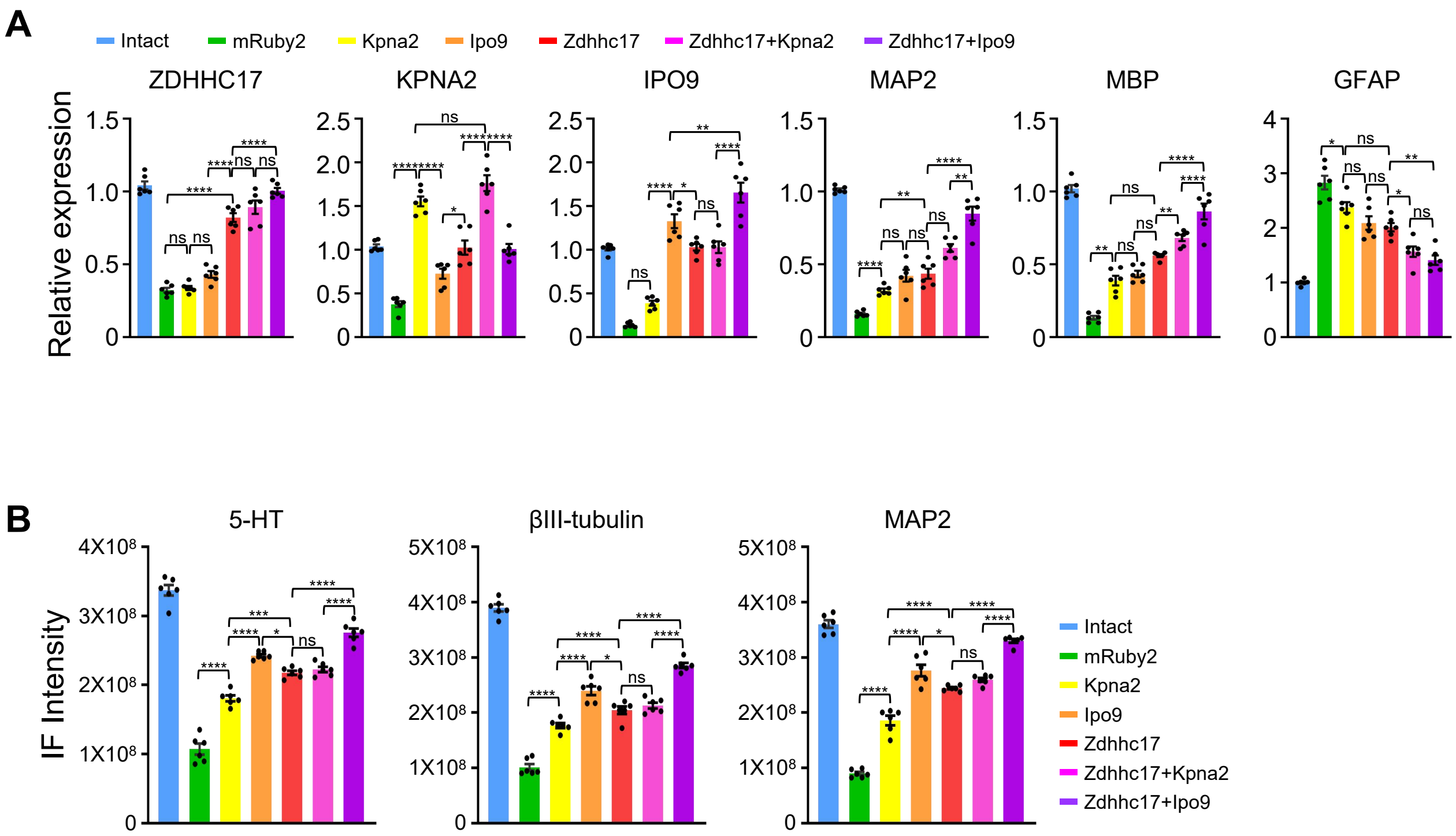
A
Intact
mRuby2
Kpna2
Ipo9
Zdhhc17
Zdhhc17+Kpna2
Zdhhc17+Ipo9
ZDHHC17
KPNA2
IPO9
MAP2
MBP
GFAP
Relative expression
B
5-HT
βIII-tubulin
MAP2
IF Intensity
Intact
mRuby2
Kpna2
Ipo9
Zdhhc17
Zdhhc17+Kpna2
Zdhhc17+Ipo9

Figure S6

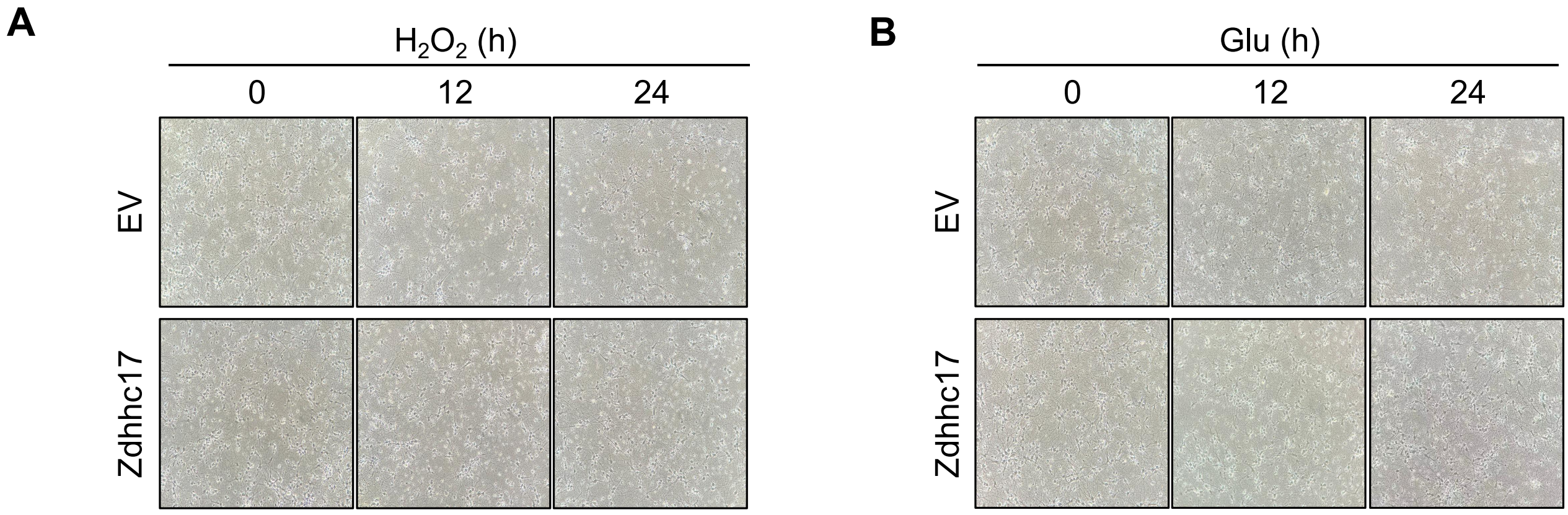

Figure S7

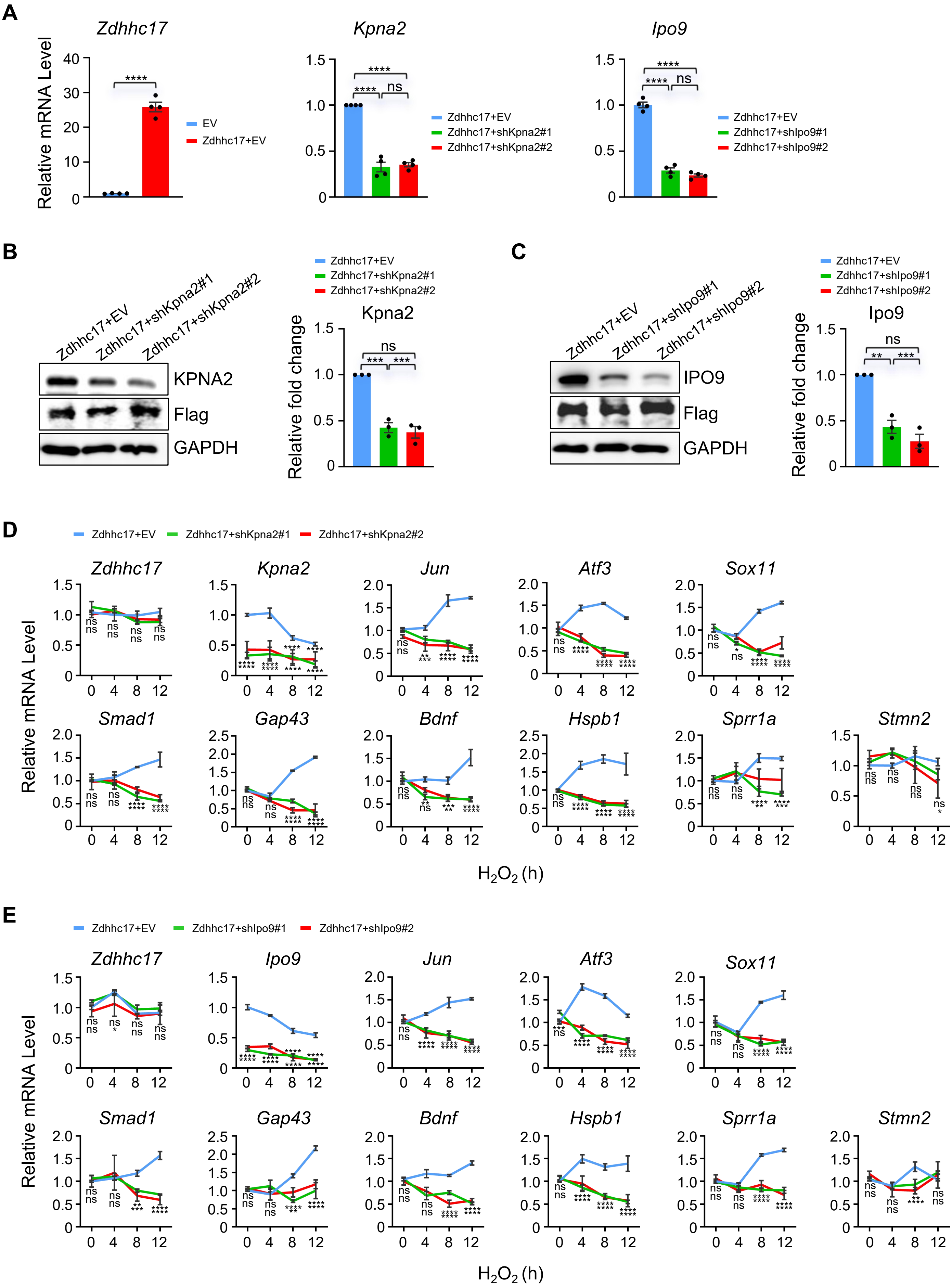

Figure S8 continued

F

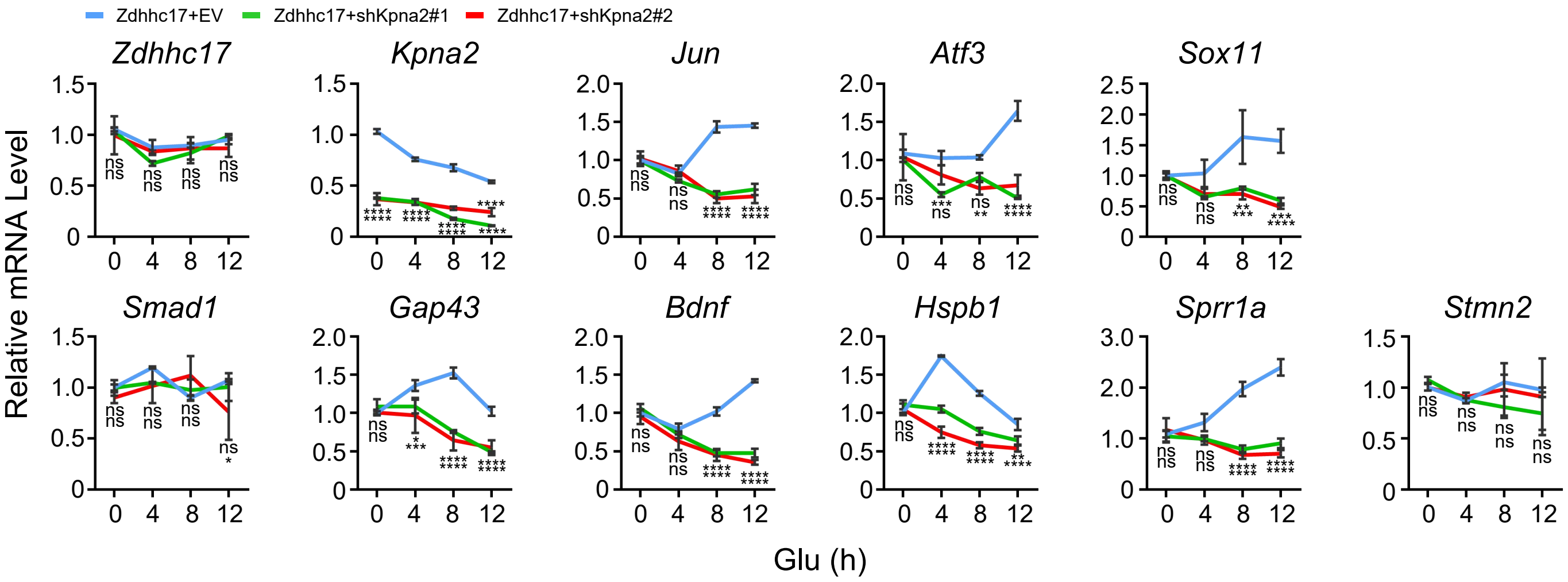

Zdhhc17+EV
Zdhhc17+shKpna2#1
Zdhhc17+shKpna2#2
Zdhhc17
Kpna2
Jun
Atf3
Sox11
Smad1
Gap43
Bdnf
Hspb1
Sprr1a
Stmn2
Relative mRNA Level
Glu (h)

G

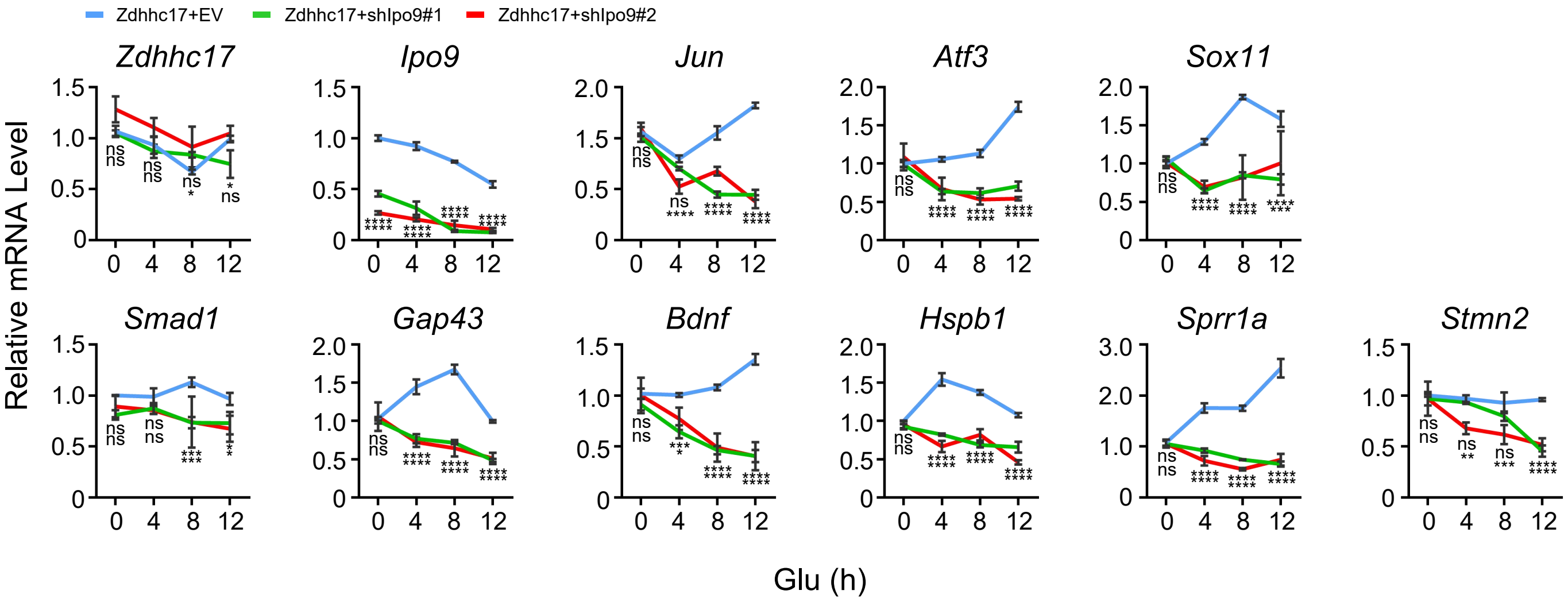

Zdhhc17+EV
Zdhhc17+shIpo9#1
Zdhhc17+shIpo9#2
Zdhhc17
Ipo9
Jun
Atf3
Sox11
Smad1
Gap43
Bdnf
Hspb1
Sprr1a
Stmn2
Relative mRNA Level
Glu (h)

Figure S8
