# Supplementary material for: Palmitoyl Acyltransferase Zdhhc17 Promotes Functional Recovery After Spinal Cord Injury by Targeting the Nuclear Transport Factors Kpna2 and Ipo9
Source: Adv Sci (Weinh). 2026 Sep 11:e77724. Online ahead of print. doi: 10.1002/advs.77724 (PMC13564187; doi:10.1002/advs.77724)
Supplement: Supplementary file 2 [file ADVS-9999-e77724-s002.pdf]

Supplementary information

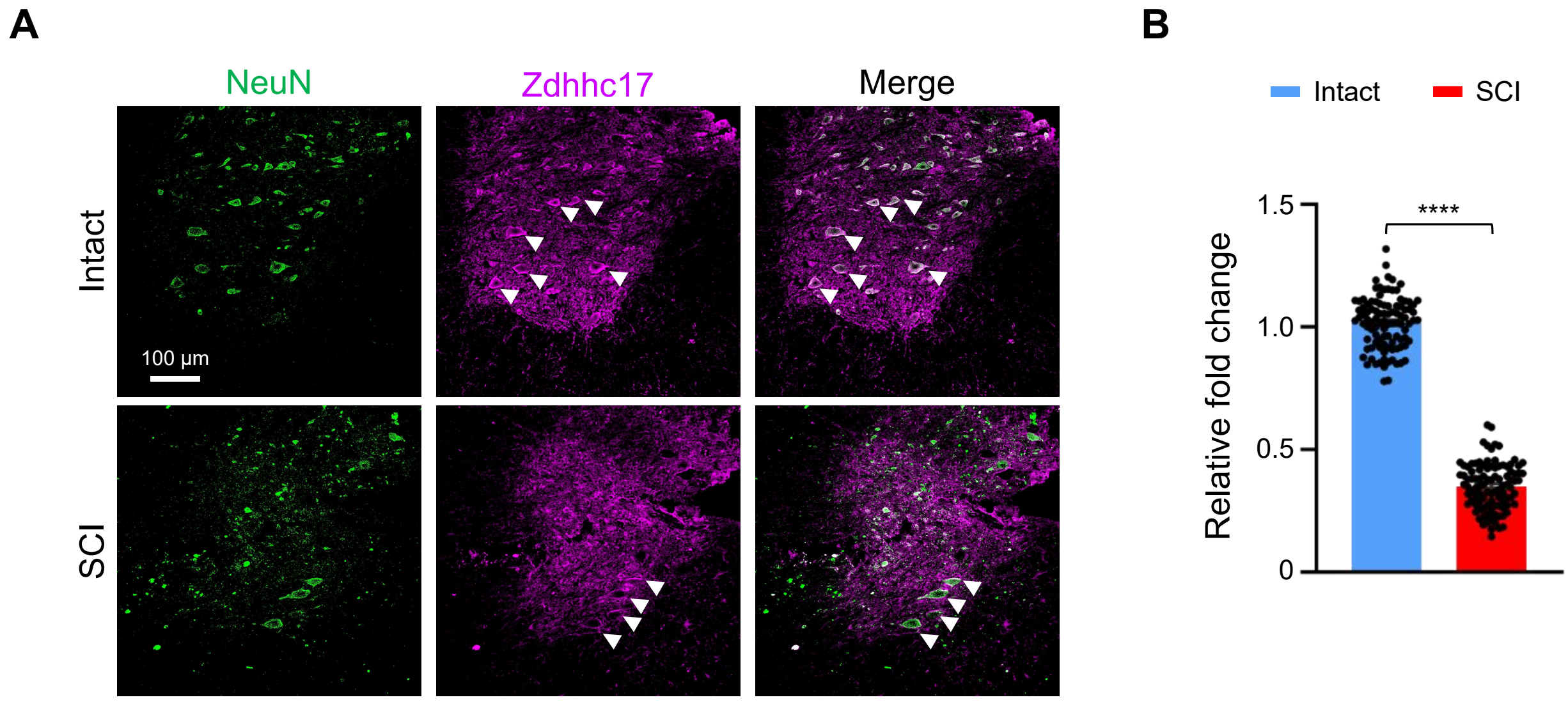
A
NeuN
Zdhhc17
Merge
Intact
SCI
100 μm
B
Intact
SCI
Relative fold change
1.5
1.0
0.5
0
****

Figure S1

Figure S1. SCI reduces Zdhhc17 expression in spinal neurons.

(A) Representative IF images of NeuN and Zdhhc17 in transverse spinal cord sections from the Intact and SCI groups at 4 wpi. Triangles indicate representative Zdhhc17-positive neurons.

(B) Quantification of relative Zdhhc17 fluorescence intensity per neuron. Data are presented as mean ± SEM (n = 100). ****$p < 0.0001$ by unpaired Student's t-test.

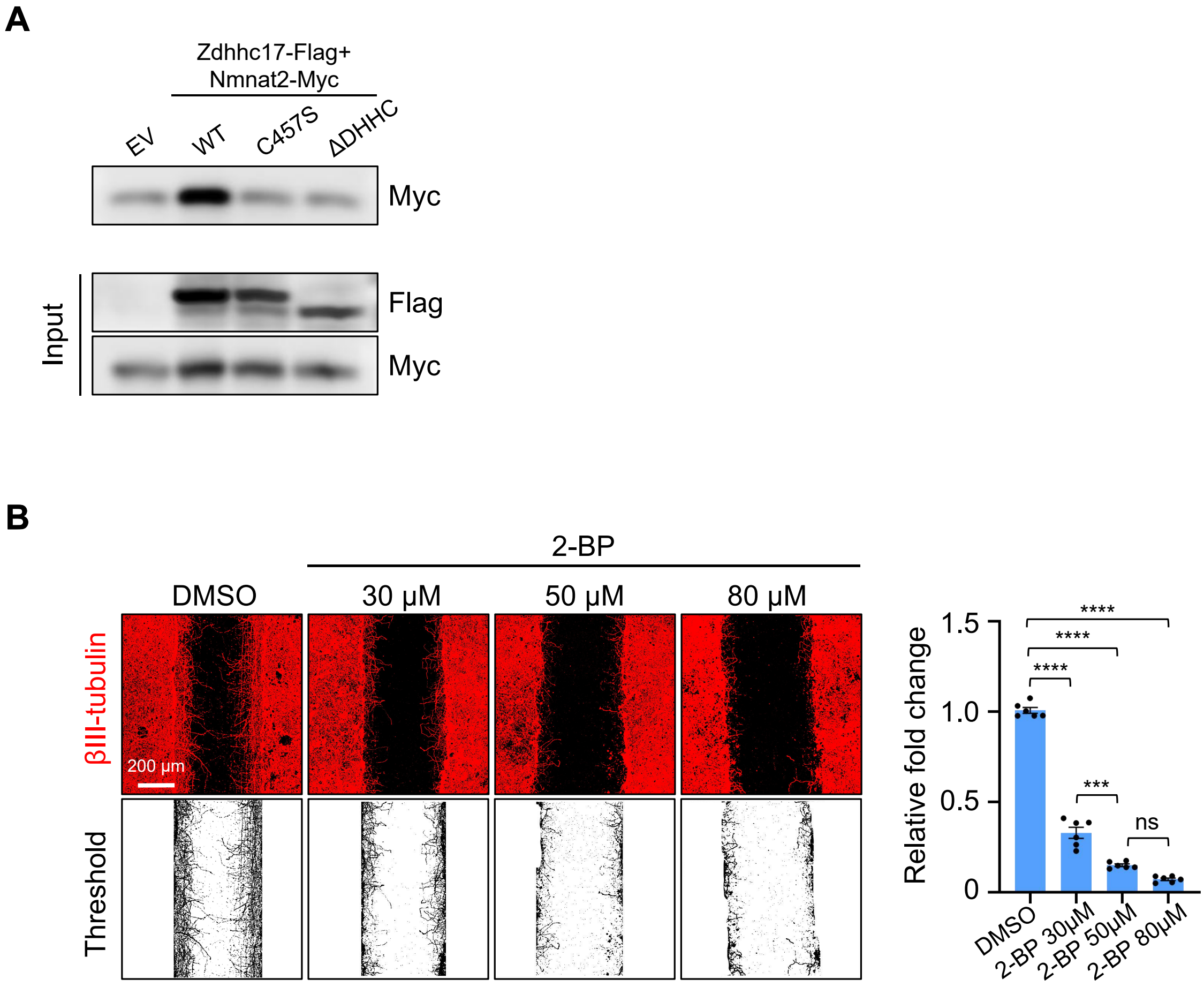
A
Zdhhc17-Flag+
Nmnat2-Myc
EV
WT
C457S
ΔDHHC
Myc
Input
Flag
Myc
B
2-BP
DMSO
30 μM
50 μM
80 μM
βIII-tubulin
200 μm
Threshold
Relative fold change
1.5
1.0
0.5
0
****
****
****
***
ns
DMSO
2-BP 30μM
2-BP 50μM
2-BP 80μM

Figure S2

Figure S2. Zdhhc17 functions as a PAT.

(A) Acyl-RAC assay verifying Zdhhc17 enzymatic mutants in HEK293T cells co-expressing Myc-tagged Nmnat2.

(B) Representative images showing the dose effect of 2-bromopalmitate (2-BP) on scratch-injured cortical cultures. Cultures were immunostained for βIII-tubulin, and the corresponding ImageJ-thresholded versions are shown. Right panel, quantification of βIII-tubulin intensity in the scratched area. Data are presented as mean ± SEM (n = 6). ns, not significant; ***$p < 0.001$; ****$p < 0.0001$ by one-way ANOVA.

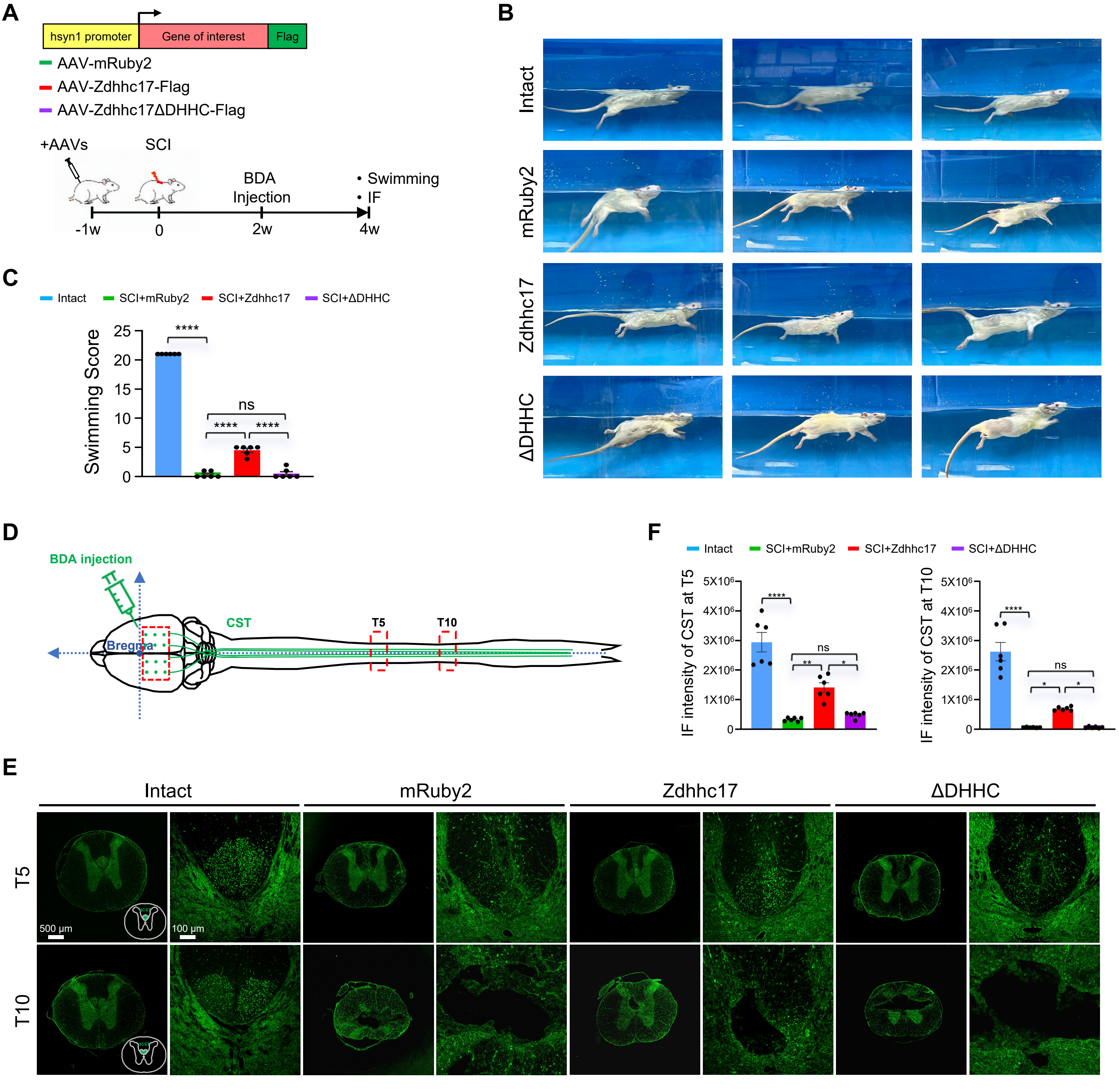
A
hsyn1 promoter
Gene of interest
Flag
AAV-mRuby2
AAV-Zdhhc17-Flag
AAV-Zdhhc17ΔDHHC-Flag
+AAVs
SCI
BDA Injection
• Swimming
• IF
-1w
0
2w
4w
B
Intact
mRuby2
Zdhhc17
ΔDHHC
C
Intact
SCI+mRuby2
SCI+Zdhhc17
SCI+ΔDHHC
Swimming Score
****
ns
D
BDA injection
Bregma
CST
T5
T10
F
IF intensity of CST at T5
IF intensity of CST at T10
E
T5
T10
500 μm
100 μm

Figure S3

Figure S3. Zdhhc17 improves swimming performance and CST axon regeneration after SCI.

(A) Schematic of the experimental design.

(B) Representative images of rat swimming performance in groups with indicated treatments.

(C) Swimming performance scores of rats with indicated treatments. Data are presented as mean ± SEM (n = 6). ns, not significant; ****$p < 0.0001$ by one-way ANOVA comparing AAV-Zdhhc17 with other groups.

(D) Schematic of BDA anterograde tracing. BDA was injected into the sensorimotor cortex, and labeled CST fibers were examined at T5 (rostral to the lesion) and T10 (the lesion site) spinal levels at 4 wpi.

(E) Representative images of BDA-labeled CST fibers in transverse spinal cord sections from the indicated groups. The lower right schematic illustrates CST-specific BDA signals in the intact group.

(F) Quantification of CST-specific BDA signal intensity in the indicated groups at T5 (left panel) and T10 (right panel) levels. Data are presented as mean ± SEM (n = 6 per group). ns, not significant; *$p < 0.05$; **$p < 0.01$; ****$p < 0.0001$ by one-way ANOVA.

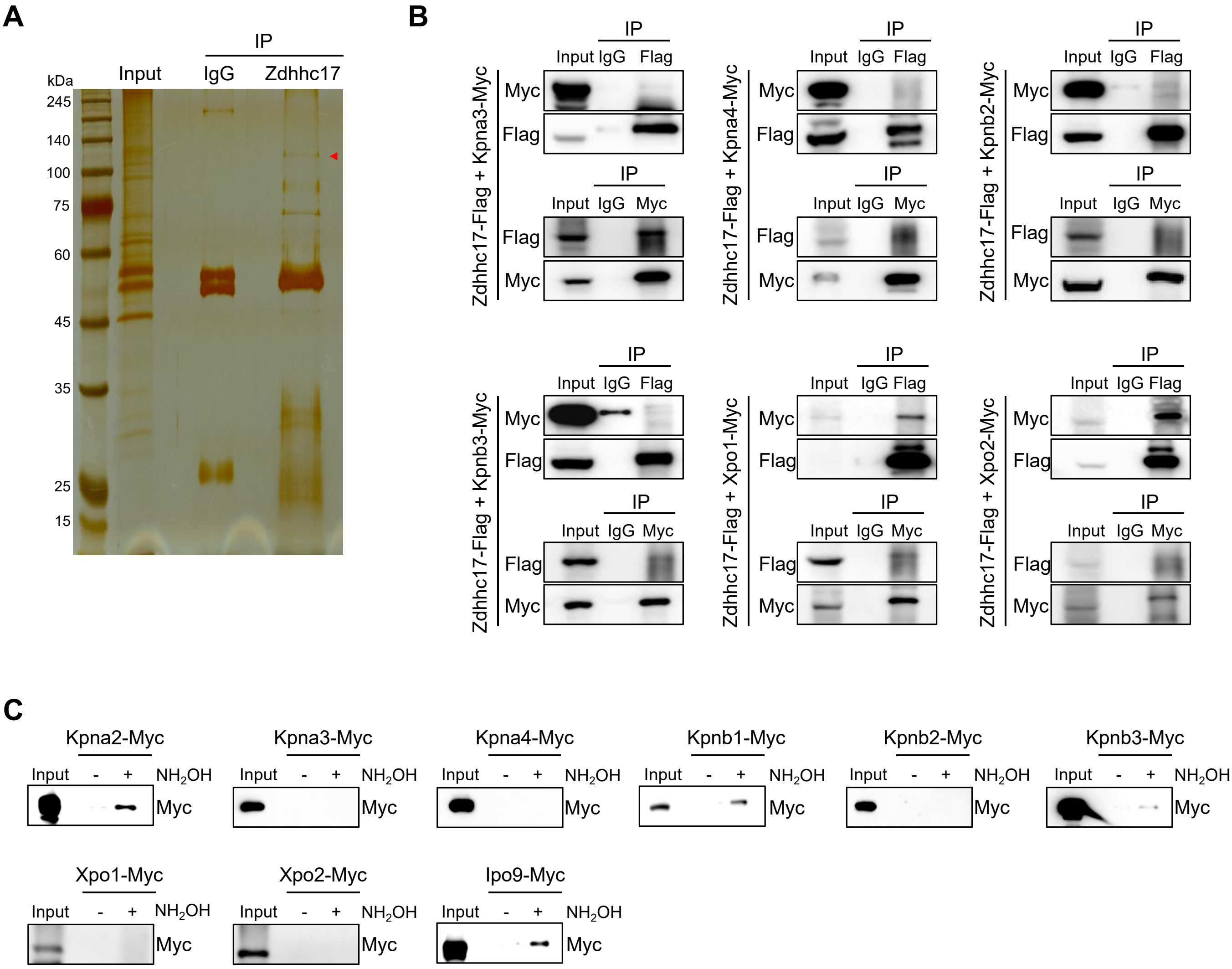
A
IP
kDa
Input
IgG
Zdhhc17
245
140
100
75
60
45
35
25
15
B
Zdhhc17-Flag + Kpna3-Myc
Zdhhc17-Flag + Kpna4-Myc
Zdhhc17-Flag + Kpnb2-Myc
Zdhhc17-Flag + Kpnb3-Myc
Zdhhc17-Flag + Xpo1-Myc
Zdhhc17-Flag + Xpo2-Myc
IP
Input
IgG
Flag
Myc
C
Kpna2-Myc
Kpna3-Myc
Kpna4-Myc
Kpnb1-Myc
Kpnb2-Myc
Kpnb3-Myc
Xpo1-Myc
Xpo2-Myc
Ipo9-Myc
Input
-
+
NH2OH
Myc

Figure S4

Figure S4. Zdhhc17 interacts with numerous Kaps.

(A) Representative silver staining of Zdhhc17-coimmuoprecipitated proteins from rat cortical neurons under native conditions. IgG IP served as a negative control. Red arrow indicates the Zdhhc17 band.

(B) Reciprocal IP-WB validation of the interactions between Zdhhc17 and six Kaps in HEK293T cells.

(C) Acyl-RAC assay detecting palmitoylation of the nine Kaps in HEK293T cells.

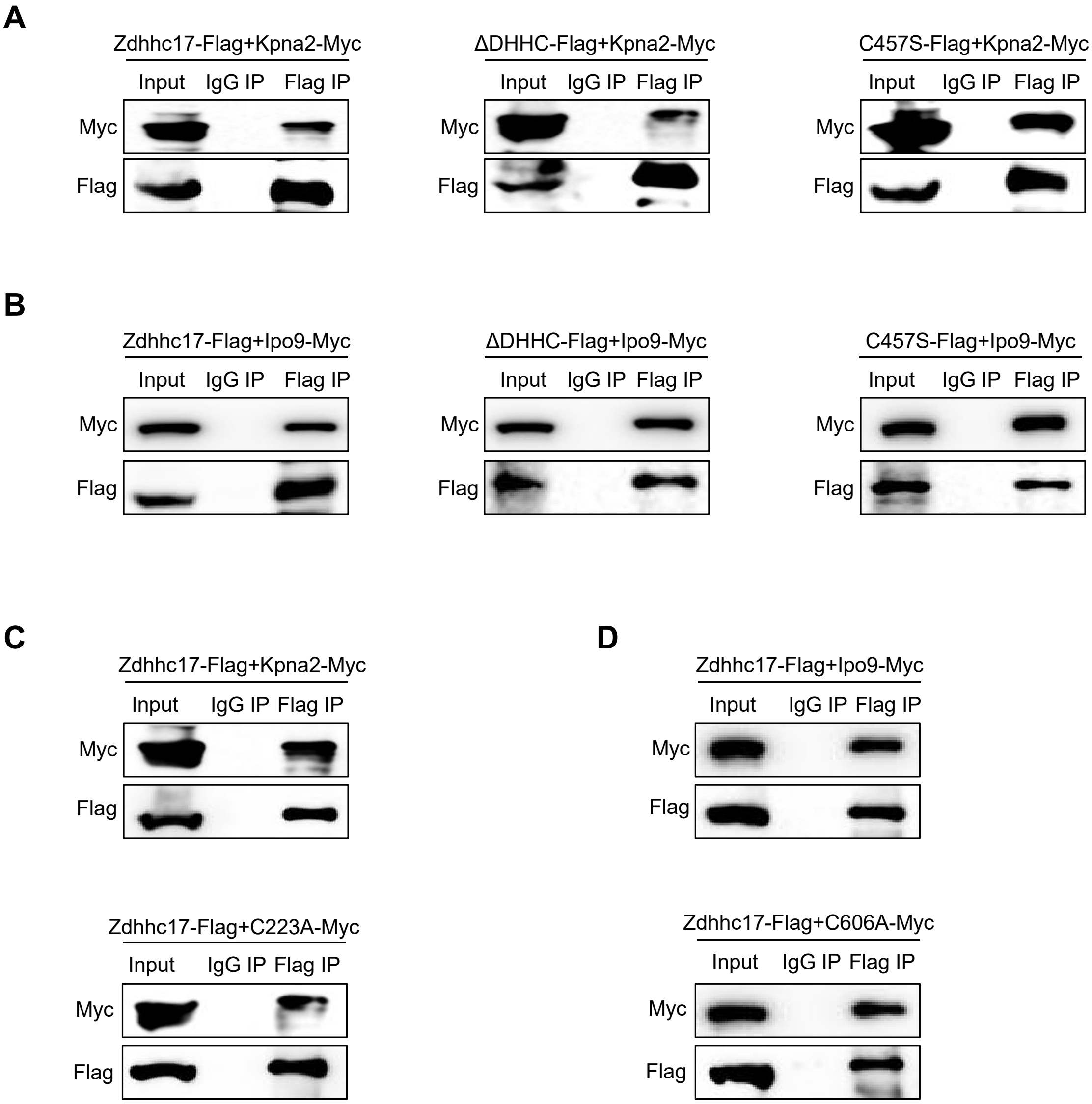
A
Zdhhc17-Flag+Kpna2-Myc
ΔDHHC-Flag+Kpna2-Myc
C457S-Flag+Kpna2-Myc
Input
IgG IP
Flag IP
Myc
Flag
B
Zdhhc17-Flag+Ipo9-Myc
ΔDHHC-Flag+Ipo9-Myc
C457S-Flag+Ipo9-Myc
C
Zdhhc17-Flag+Kpna2-Myc
Zdhhc17-Flag+C223A-Myc
D
Zdhhc17-Flag+Ipo9-Myc
Zdhhc17-Flag+C606A-Myc

Figure S5

Figure S5. Mutations in the catalytic domain of Zdhhc17 or at the palmitoylation sites of Kpna2 and Ipo9 do not disrupt their mutual interactions.

(A, B) IP-WB validation of the interaction of WT and mutant Zdhhc17 with Kpna2 (A) and Ipo9 (B) in HEK293T cells.

(C) IP–WB showing Zdhhc17 binds to both WT Kpna2 and the palmitoylation-site mutant Kpna2 C223A.

(D) IP-WB showing Zdhhc17 binds to both WT Ipo9 and the palmitoylation- site mutant Ipo9 C606A.

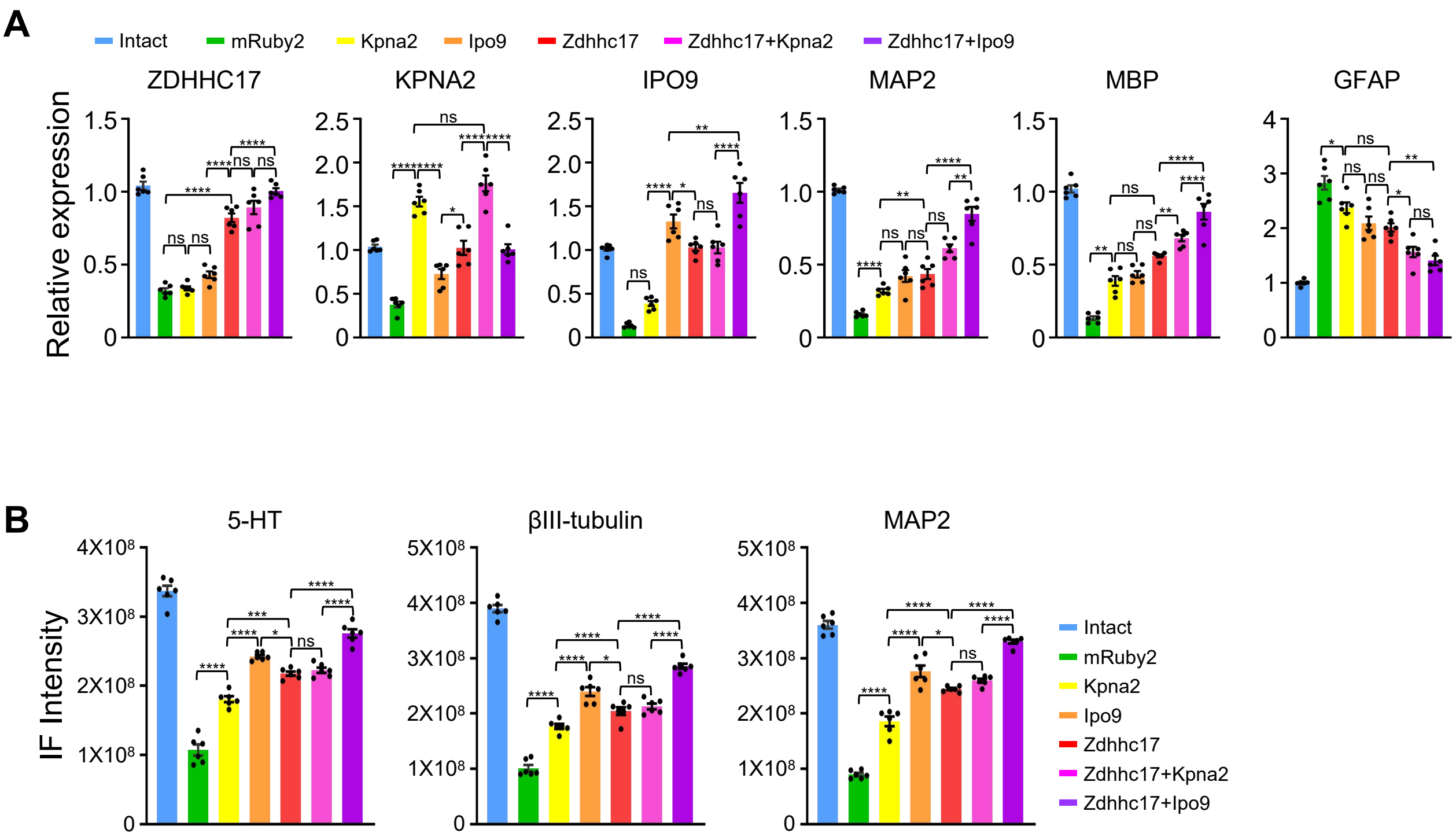
A
Intact
mRuby2
Kpna2
Ipo9
Zdhhc17
Zdhhc17+Kpna2
Zdhhc17+Ipo9
ZDHHC17
KPNA2
IPO9
MAP2
MBP
GFAP
Relative expression
B
5-HT
βIII-tubulin
MAP2
IF Intensity

Figure S6

Figure S6. Kpna2 and Ipo9 mimic the therapeutic effect of Zdhhc17 on SCI repair.

(A) Quantification of the indicated protein expression levels in Figure 7B. The color key is consistent across all panels. Data are presented as the mean ± SEM; n = 3/group. ns, not significant; *$p < 0.05$; **$p < 0.01$; ***$p < 0.001$; ****$p < 0.0001$ by one-way ANOVA.

(B) Quantification of the indicated immunoreactive intensity in Figure 7J. Data are presented as mean ± SEM (n = 6 per group). ns, not significant; *$p < 0.05$; ***$p < 0.001$; ****$p < 0.0001$ by one-way ANOVA.

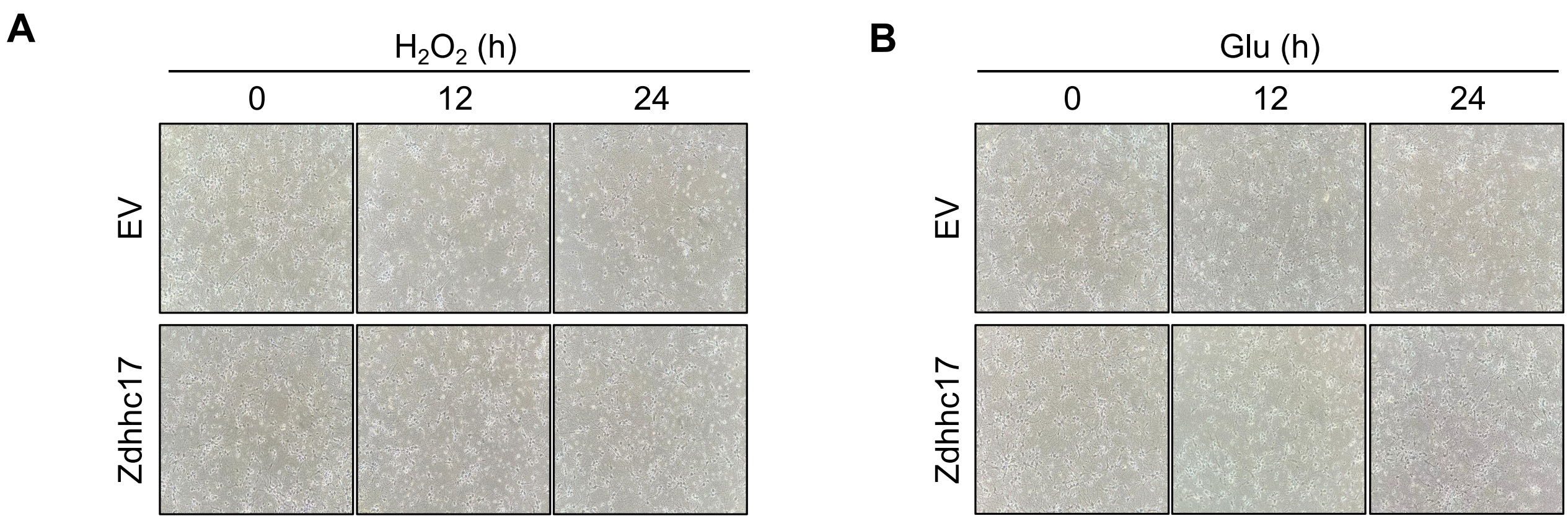

Figure S7

Figure S7. H_2O_2-induced oxidative stress and glutamate- induced excitotoxicity models in primary cortical neurons.

(A, B) Representative phase-contrast images showing neurons transduced with lentivirus expressing EV (upper panels) or Zdhhc17 (lower panels) at 0, 12 and 24 h after treatment with 300 μM H_2O_2 (A) or 4 μM Glutamate (B).

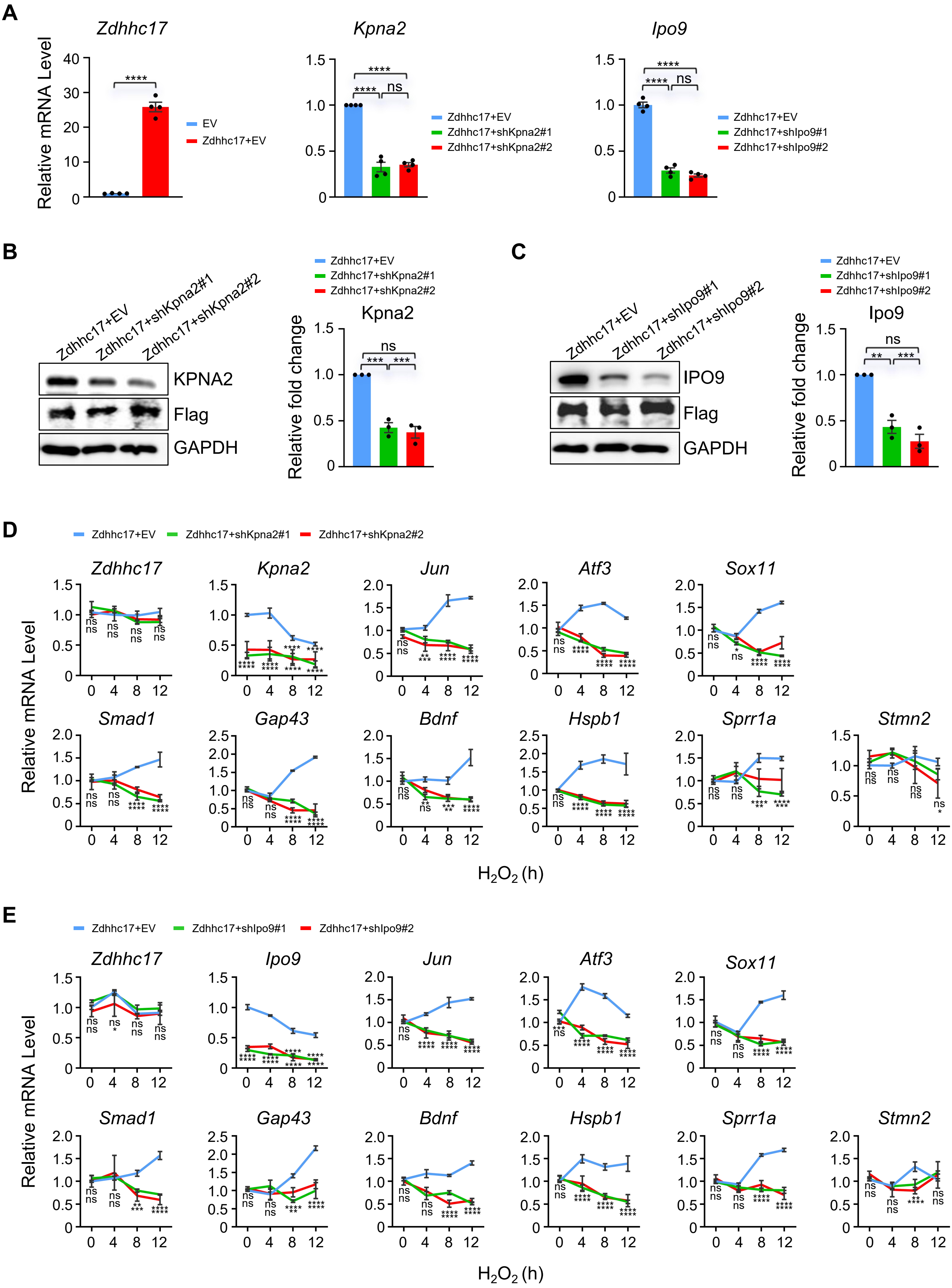

Figure S8 continued

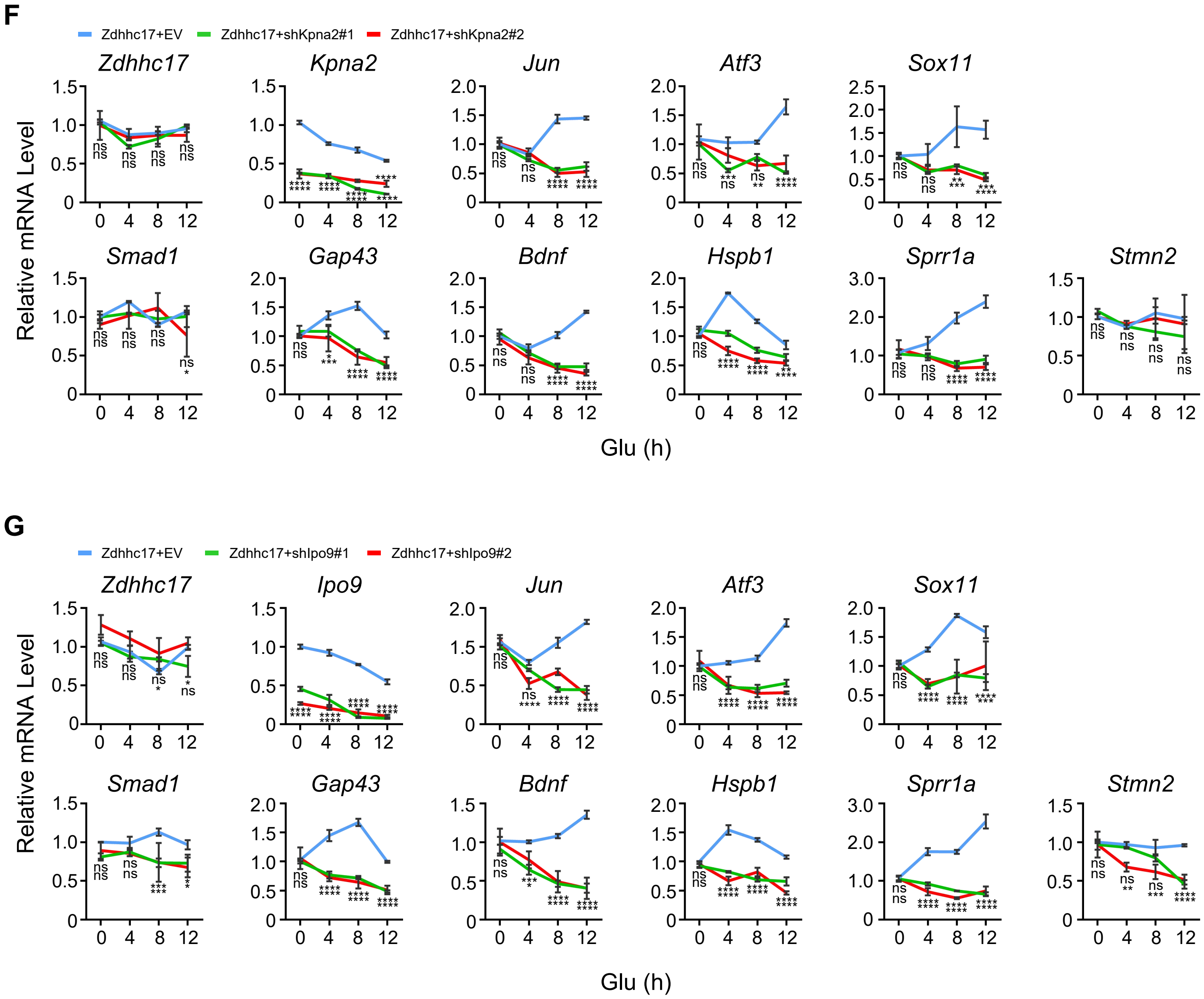
F
Zdhhc17+EV
Zdhhc17+shKpna2#1
Zdhhc17+shKpna2#2
Zdhhc17
Kpna2
Jun
Atf3
Sox11
Smad1
Gap43
Bdnf
Hspb1
Sprr1a
Stmn2
Relative mRNA Level
0
4
8
12
Glu (h)
G
Zdhhc17+EV
Zdhhc17+shIpo9#1
Zdhhc17+shIpo9#2
Zdhhc17
Ipo9
Jun
Atf3
Sox11
Smad1
Gap43
Bdnf
Hspb1
Sprr1a
Stmn2
Relative mRNA Level
0
4
8
12
Glu (h)

Figure S8

Figure S8. Knockdown of Kpna2 or Ipo9 prior to injury attenuates Zdhhc17-mediated transcriptional activation of pro-regenerative genes.

(A) Real-time PCR analysis of Zdhhc17, Kpna2, and Ipo9 mRNA levels in primary neurons co-expressing Zdhhc17 and shRNAs targeting either Kpna2 or Ipo9. Data are presented as mean ± SEM (n = 4). ns, not significant; ****$p < 0.0001$ by one-way ANOVA.

(B, C) WB analysis of Kpna2 (B) and Ipo9 (C) protein levels in primary neurons co-expressing Flag-tagged Zdhhc17 and shRNAs targeting either Kpna2 or Ipo9. Right panels show quantification of the indicated protein levels. Data are presented as mean ± SEM (n = 3). ns, not significant; **$p < 0.01$; ***$p < 0.001$; ****$p < 0.0001$ by one-way ANOVA.

(D-G) Real-time PCR analysis of Zdhhc17, Kpna2, Ipo9, and pro-regenerative genes mRNA levels in primary cortical neurons co-expressing Zdhhc17 and shRNAs targeting either Kpna2 (D, F) or Ipo9 (E, G), following a time-course H_2O_2 (D, E) or glutamate (F, G) treatment. Data are presented as mean ± SEM (n = 4). ns, not significant; *$p < 0.05$; **$p < 0.01$; ***$p < 0.001$; ****$p < 0.0001$ by two-way ANOVA comparing Zdhhc17+EV with other groups.

Table S1. Oligos

Oligo Name (Rat)	Sequence (5' - 3')
Primer list for PCR	
Fwd_NMNAT2	GGATCTATTTCCGGTGAATTCATGACCGAGACCACAAAGACCC
Rev_NMNAT2	ATGGTCCTTGTAGTCACTAGTGCCCGAGGCATTGATGTACA
Fwd_Zdhhc17	GGATCTATTTCCGGTGAATTCATGGCGGACGGCCCGGAT
Rev_Zdhhc17	ATGGTCCTTGTAGTCACTAGTCTACACCAGCTGGTACCCAGATC
Fwd_Zdhhc17ΔDHHC	ACCGGCAGTCTGGACCTCAGTTTCTTGCTTTTTATGATCTGC
Rev_Zdhhc17ΔDHHC	GCAGATCATAAAAAGCAAGAAACTGAGGTCCAGACTGCCGGT
Fwd_Zdhhc17.C457S	ATCACCATAGCCCATGGGTGGGTAACTGTGTA
Rev_Zdhhc17.C457S	CCATGGGCTATGGTGATCAAATTTCGCTATACAG
Fwd_Zdhhc17.shRNA#1	CCGGGCCATCAATAACAGAATAGATCTCGAGATCTATTCTGTTAT TGA TGGCTTTTT
Rev_Zdhhc17.shRNA#1	AATTAAAAAGCCATCAATAACAGAATAGATCTCGAGATCTATT CTGT TATTGATGGC
Fwd_Zdhhc17.shRNA#2	CCGGCCTTTAATGTGGGCAGCTTATCTCGAGATAAGCTGCCCA CATT AAAGGTTTTT
Rev_Zdhhc17.shRNA#2	AATTAAAAACCTTTAATGTGGGCAGCTTATCTCGAGATAAGCTG CCC ACATTAAAGG
Fwd_Zdhhc17.shRNA#3	CCGGCATTAAAGCTACAGAAGAACACTCGAGTGTTCTTCTGTA GCT TTAATGTTTTT
Rev_Zdhhc17.shRNA#3	AATTAAAAACATTAAAGCTACAGAAGAACACTCGAGTGTTCTT CTG TAGCTTTAATG
Fwd_Zdhhc17.shRNA#4	CCGGATGATCTGCTGGATGATTTATCTCGAGATAAATCATCCAG CAG ATCATTTTTT
Rev_Zdhhc17.shRNA#4	AATTAAAAAATGATCTGCTGGATGATTTATCTCGAGATAAATCAT CCA GCAGATCAT
Fwd_Zdhhc17.shRNA#5	CCGGATGAATGCCAGGAGATACAAGCTCGAGCTTGTATCTCCTG GCATTCATTTTTT
Rev_Zdhhc17.shRNA#5	AATTAAAAAATGAATGCCAGGAGATACAAGCTCGAGCTTGTAT CTCC TGGCATTCAT
Fwd_Kpna2	GGATCTATTTCCGGTGAATTCATGTCCACCAATGAGAATGCTAA C
Rev_Kpna2	GATGAGTTTCTGCTCACTAGTGAAGTTAAAGGTCCCGGGAGC
Fwd_Kpna3	GGATCTATTTCCGGTGAATTCATGGCCGAGAACCCCGGC

Rev_Kpna3	GATGAGTTTCTGCTCACTAGTGAAATTAAATTCTTTTGTTTGAA GGT TG
Fwd_Kpna4	GGATCTATTTCCGGTGAATTCATGGCGGACAACGAGAAATTG
Rev_Kpna4	GATGAGTTTCTGCTCACTAGTAAACTGGAACCCCTCTGTTGG
Fwd_Kpnb1	GGATCTATTTCCGGTGAATTCATGGAGCTCATAACCATCCTCG
Rev_Kpnb1	GATGAGTTTCTGCTCACTAGTAGCTTGGTTCTTCAGTTTCCTCA
Fwd_Kpnb2	GGATCTATTTCCGGTGAATTCATGGAGTATGAGTGGAAACCTGA CG
Rev_Kpnb2	GATGAGTTTCTGCTCACTAGTAACACCATAAAAAGCCGCAAGA
Fwd_Kpnb3	GGATCTATTTCCGGTGAATTCATGGCGGCGGCCGCGGCG
Rev_Kpnb3	GATGAGTTTCTGCTCACTAGTGGCAGAGTTCAGGAGCTCCTG
Fwd_Ipo9	GGATCTATTTCCGGTGAATTCATGGCGGCGGCGGCGGCA
Rev_Ipo9	GATGAGTTTCTGCTCACTAGTGATGCCGATAGTCTGTAGGACCC
Fwd_Xpo1	GGATCTATTTCCGGTGAATTCATGCCAGCAATTATGACAATGTTA G
Rev_Xpo1	GATGAGTTTCTGCTCACTAGTGTCACACATTTCTTCTGGGATTTC A
Fwd_Xpo2	GGATCTATTTCCGGTGAATTCATGGAGCTCAGCGATGCCA
Rev_Xpo2	GATGAGTTTCTGCTCACTAGTAAGGTGTGAACCAGCATCCAGC
Fwd_Kpna2.shRNA#1	CCGGGCTGGTTTGATCCCAAAATTTCTCGAGAAATTTTGGGAT CAAACCAGCTTTTT
Rev_Kpna2.shRNA#1	AATTAAAAAGCTGGTTTGATCCCAAAATTTCTCGAGAAATTTTG GGATCAAACCAGC
Fwd_Kpna2.shRNA#2	CCGGGAGGACATTGTTAAAGGCATACTCGAGTATGCCTTTAAC AATGTCCTCTTTTT
Rev_Kpna2.shRNA#2	AATTAAAAAGAGGACATTGTTAAAGGCATACTCGAGTATGCCT TTAACAATGTCCTC
Fwd_Kpna2.shRNA#3	CCGGCCGGCTTAACAGATTCAAGAACTCGAGTTCTTGAATCTGT TAAGCCGGTTTTT
Rev_Kpna2.shRNA#3	AATTAAAAACCGGCTTAACAGATTCAAGAACTCGAGTTCTTGA ATCTGTTAAGCCGG
Fwd_Kpna2.shRNA#4	CCGGCATGGGCTATAACCAACTACACTCGAGTGTAGTTGGTTAT AGCCCATGTTTTT
Rev_Kpna2.shRNA#4	AATTAAAAACCGGCTTAACAGATTCAAGAACTCGAGTTCTTGA ATCTGTTAAGCCGG
Fwd_Kpna2.shRNA#5	CCGGCCTGGACACTTTCAAACCTCTCTCGAGAGAGGTTTGAAA GTGTCCAGGTTTTT
Rev_Kpna2.shRNA#5	AATTAAAAACCTGGACACTTTCAAACCTCTCTCGAGAGAGGTTT GAAAGTGTCCAGG
Fwd_Ipo9.shRNA#1	CCGGCAAGAAGCTGAGCAGACGAAACTCGAGTTTCGTCTGCT CAGCTTCTTGTTTTT

Rev_Ipo9.shRNA#1	AATTAAAAACAAGAAGCTGAGCAGACGAAACTCGAGTTTCGTCTGCTCAGCTTCTTG
Fwd_Ipo9.shRNA#2	CCGGCCAAGATGATTCCAATGATATCTCGAGATATCATTGGAATCATCTTGGTTTTT
Rev_Ipo9.shRNA#2	AATTAAAAACCAAGATGATTCCAATGATATCTCGAGATATCATTGGAATCATCTTGG
Fwd_Ipo9.shRNA#3	CCGGCACTCAGCTGGAACCTCTGTTCTCGAGAACAGAGGTTCCAGCTGAGTGTTTTT
Rev_Ipo9.shRNA#3	AATTAAAAACACTCAGCTGGAACCTCTGTTCTCGAGAACAGAGGTTCCAGCTGAGTG
Fwd_Ipo9.shRNA#4	CCGGAGCATCCTGGATGGCCTAATTCTCGAGAATTAGGCCATCCAGGATGCTTTTT
Rev_Ipo9.shRNA#4	AATTAAAAAAGCATCCTGGATGGCCTAATTCTCGAGAATTAGGCCATCCAGGATGCT
Fwd_Ipo9.shRNA#5	CCGGTTGACTACAGTAGTTCGAAATCTCGAGATTTCGAACTACTGTAGTCAATTTTT
Rev_Ipo9.shRNA#5	AATTAAAAATTGACTACAGTAGTTCGAAATCTCGAGATTTCGAACTACTGTAGTCAA
Fwd_Zdhhc17 (forAAV)	GAGCGCAGTCGAGAAGGTACCGCCACCATGGCGGACGGCCCGGAT
Fwd_Kpna2 (forAAV)	GAGCGCAGTCGAGAAGGTACCGCCACCATGTCCACCAATGAGAATGCTAAC
Fwd_Ipo9 (forAAV)	GAGCGCAGTCGAGAAGGTACCGCCACCATGGCGGCGGCGGCGGCA
Primer list for qPCR	
Fwd_28S	TGTGGTTTCGCTGGATAGTAGGT
Rev_28S	TTCGTATTGCGCCGCTAGA
Fwd_Zdhhc17	CTTCCTTGCCAACAGCGTTGCT
Rev_Zdhhc17	TGAGGTCCAGACTTCCAGTCTC
Fwd_Kpna2	GCTCATTCCCTGATGACGCT
Rev_Kpna2	GAAAGCAGTTTCCGAGCAGC
Fwd_Kpnb1	TAATCCAGGGCATGCGGAAG
Rev_Kpnb1	GCAGCCACTCTTACCCTTGTA
Fwd_Ipo9	CTGGAGGTGACAGAGGAATTTG
Rev_Ipo9	ACTGGAGCGCACTTTGCTTA
Fwd_Jun	TGTGCCCCAAGAACGTGAC
Rev_Jun	CCGGGTTGAAGTTGCTGAG
Fwd_Smad1	CTCATGTCATTTATTGCCGTGTG

Rev_Smad1	CGCTTATAGTGGTAGGGGTTGA
Fwd_Sprr1a	CAAGGCACCTGAGCCCTGCAA
Rev_Sprr1a	AGGCTCTGGTGCCTTAGGTTGG
Fwd_Hspb1	GCTCACAGTGAAGACCAAGGAAG
Rev_Hspb1	TGAAGCACCGAGAGATGTAGCC
Fwd_Stmn2	CAGAAGCTCCACGAACTCTAGC
Rev_Stmn2	TGCTTCAGCACCTGAGCCTCTT
Fwd_Sox11	GGACCTGGATTCCTTCAGTGAG
Rev_Sox11	GTGAACACCAGGTCGGAGAAGT
Fwd_Gap43	GAGGAGCCTAAACAAGCCGA
Rev_Gap43	GGCTTCATCTACAGCTTCTTTCT
Fwd_Jun	TGTGCCCCAAGAACGTGAC
Rev_Jun	CCGGGTTGAAGTTGCTGAG
Fwd_Bdnf	GCTTGGAGAAGGAAACCGCC
Rev_Bdnf	CTCACCTGGTGGAACTCAGGG
Fwd_Atf3	AGCGAAGACTGGAGCAAAATG
Rev_Atf3	AGGACATCCGATGGCAAAGG

Table S2. Plasmids

Plasmids	Source
pLVX-IRES-Puro	Cat#632183, Clontech
pLKO.1 puro	Cat#8453, Addgene
pCMV-VSVG	Cat#8454, Addgene
pCMV-deltaR8.2	Cat#12263, Addgene
pAAV-pUCmini-iCAP-PHP.	Cat#103005, Addgene
pHelper	Cat#ZK736, ZomanBio
pAAV-hSyn1-mRuby2	Cat#99126, Addgene
pLKO.1 EGFP	This paper
pLVX-IRES-CMV-EGFP-puro	This paper
pLVX-IRES-CMV-Zdhhc17-Flag-puro	This paper
pLVX-IRES-CMV-Zdhhc17ΔDHHC-Flag-puro	This paper
pLVX-IRES-CMV-Zdhhc17.C457S-Flag-puro	This paper
pLKO-IRES-shZdhhc17#1-5-EGFP	This paper
pLVX-IRES-CMV-NMNAT2-Myc-puro	This paper
pLVX-IRES-CMV-Kpna2-Myc-puro	This paper
pLVX-IRES-CMV-Kpnb1-Myc-puro	This paper
pLVX-IRES-CMV-Ipo9-Myc-puro	This paper
pLVX-IRES-CMV-Kpna2.C223A-Myc-puro	This paper
pLVX-IRES-CMV-Kpna2.C467A-Myc-puro	This paper
pLVX-IRES-CMV-Ipo9.C606A-Myc-puro	This paper
pLVX-IRES-CMV-Ipo9.C639A-Myc-puro	This paper
pAAV-hSyn1-Zdhhc17-Flag	This paper
pAAV-hSyn1-Zdhhc17ΔDHHC-Flag	This paper
pAAV-hSyn1-Zdhhc17.C457S-Flag	This paper
pAAV-hSyn1--Kpna2-Myc	This paper
pAAV-hSyn1--Ipo9-Myc	This paper

Table S3. Antibodies

Antibody name	Company	Catalog No.
Anti-Myc-tag	Abmart	M20002M
Anti-Flag-tag	Abmart	M20008M
Anti-GAPDH	Abcam	ab8245
Alexa Fluor™ 594 Don anti-Rabbit IgG(H+L)	Thermo Scientific	A21207
Alexa Fluor™ 488 Goat anti-Rabbit IgG (H+L)	Thermo Scientific	A11008
Alexa Fluor™ 594 Goat anti-Mouse IgG(H+L)	Thermo Scientific	A11005
Alexa Fluor™ 488 Goat anti-Mouse IgG(H+L)	Thermo Scientific	A32723
Alexa Fluor™ 488 Donkey anti-Goat IgG (H+L)	Thermo Scientific	A11055
Alexa Fluor™ 647 Donkey anti-Goat IgG (H+L)	Thermo Scientific	A21447
Alexa Fluor™ 568 Goat anti-Chicken IgY (H+L)	Thermo Scientific	A11041
Alexa Fluor™ 488 streptavidin	Thermo Scientific	S11223
anti-Rabbit	Thermo Scientific	656120
anti-Mouse	Thermo Scientific	626520
anti-Goat	Abclonal	AS029
Normal Rabbit IgG	CST	2729
Normal Mouse IgG	CST	68860
Normal Goat IgG	R&D Systems	AB108C
anti-Kpna2 (rabbit)	Proteintech	668701
anti-Kpnb1 (mouse)	Proteintech	675971
anti-Ipo9 (mouse)	Abconal	A20901
Anti-MAP2 antibody (chicken)	Abcam	ab5392
Anti-MBP antibody (rabbit)	Proteintech	10458-1-AP
Anti-βIII-tubulin antibody (rabbit)	Abcam	ab18207
Anti-5-HT antibody (rabbit)	IMMUNOSTAR	20080
Anti-GFAP antibody (rabbit)	Proteintech	16825
Anti-Zdhhc17 antibody (rabbit)	Proteintech	30022-1-AP
Anti-Zdhhc17 antibody (goat)	Thermo Scientific	PA5-18336

Anti-Ubiquitin antibody (rabbit)	CST	58395S
Anti-Cleaved Caspase-3 antibody (rabbit)	CST	966lS
Anti-NeuN (mouse)	Abcam	ab177487
